# Prophage induction, but not production of phage particles, is required for lethal disease in a microbiome-replete murine model of enterohemorrhagic *E*. *coli* infection

**DOI:** 10.1371/journal.ppat.1007494

**Published:** 2019-01-10

**Authors:** Sowmya Balasubramanian, Marcia S. Osburne, Haley BrinJones, Albert K. Tai, John M. Leong

**Affiliations:** 1 Department of Molecular Biology and Microbiology at Tufts University School of Medicine, Boston, MA, United States of America; 2 Department of Immunology at Tufts University School of Medicine, Boston, MA, United States of America; University of Illinois, UNITED STATES

## Abstract

Enterohemorrhagic *Escherichia coli* (EHEC) colonize intestinal epithelium by generating characteristic attaching and effacing (AE) lesions. They are lysogenized by prophage that encode Shiga toxin 2 (Stx2), which is responsible for severe clinical manifestations. As a lysogen, prophage genes leading to lytic growth and *stx2* expression are repressed, whereas induction of the bacterial SOS response in response to DNA damage leads to lytic phage growth and Stx2 production both *in vitro* and in germ-free or streptomycin-treated mice. Some commensal bacteria diminish prophage induction and concomitant Stx2 production *in vitro*, whereas it has been proposed that phage-susceptible commensals may amplify Stx2 production by facilitating successive cycles of infection *in vivo*. We tested the role of phage induction in both Stx production and lethal disease in microbiome-replete mice, using our mouse model encompassing the murine pathogen *Citrobacter rodentium* lysogenized with the Stx2-encoding phage Φ*stx*_*2dact*_. This strain generates EHEC-like AE lesions on the murine intestine and causes lethal Stx-mediated disease. We found that lethal mouse infection did not require that Φ*stx*_*2dact*_ infect or lysogenize commensal bacteria. In addition, we detected circularized phage genomes, potentially in the early stage of replication, in feces of infected mice, confirming that prophage induction occurs during infection of microbiota-replete mice. Further, *C*. *rodentium* (Φ*stx*_*2dact*_) mutants that do not respond to DNA damage or express *stx* produced neither high levels of Stx2 *in vitro* or lethal infection *in vivo*, confirming that SOS induction and concomitant expression of phage-encoded *stx* genes are required for disease. In contrast, *C*. *rodentium* (Φ*stx*_*2dact*_) mutants incapable of prophage genome excision or of packaging phage genomes retained the ability to produce Stx *in vitro*, as well as to cause lethal disease in mice. Thus, in a microbiome-replete EHEC infection model, lytic induction of Stx-encoding prophage is essential for lethal disease, but actual phage production is not.

## Author summary

Enterohemorrhagic *Escherichia coli* (EHEC), a food-borne pathogen that produces Shiga toxin, is associated with serious disease outbreaks worldwide, including over 390 food poisoning outbreaks in the U.S. in the last two decades. Humans acquire EHEC by ingesting contaminated food or water, or through contact with animals or their environment. Infection and toxin production may result in localized hemorrhagic colitis, but may progress to life-threatening systemic hemolytic uremic syndrome (HUS), the leading cause of kidney failure in children. Treatment for EHEC or HUS remains elusive, as antibiotics have been shown to exacerbate disease. Shiga toxin genes reside on a dormant bacterial virus present in the EHEC genome, but are expressed when the virus is induced to leave its dormant state and begin to replicate. Extensive virus replication has been thought necessary to produce sufficient toxin to cause disease. Using viral and bacterial mutants in our EHEC disease mouse model, we showed that whereas an inducing signal needed to begin viral replication was essential for lethal disease, virus production was not: sufficient Shiga toxin was produced to cause lethal mouse disease, even without viral replication. Future analyses of EHEC-infected human samples will determine whether this same phenomenon applies, potentially directing intervention strategies.

## Introduction

Shiga toxin-producing *Escherichia coli* (STEC) is a food-borne zoonotic agent associated with worldwide disease outbreaks that pose a serious public health concern. Enterohemorrhagic *Escherichia coli* (EHEC), a subset of STEC harboring specific virulence factors that promote a specific mode of colonization of the intestinal epithelium (see below), is acquired by humans by ingestion of contaminated food or water, or through contact with animals or their environment. EHEC serotype O157:H7 is a major source of *E*. *coli* food poisoning in the United States, accounting for more than 390 outbreaks in the last two decades [1–5]. EHEC infection usually presents as localized hemorrhagic colitis, and may progress to the life-threatening systemic hemolytic uremic syndrome (HUS), characterized by the triad of hemolytic anemia, thrombocytopenia, and renal failure [5, 6]. HUS is the leading cause of renal failure in children [7].

EHEC, along with enteropathogenic *E*. *coli* and *Citrobacter rodentium* belong to the family of bacteria known as attaching and effacing (AE) pathogens that are capable of forming pedestal-like structures beneath bound bacteria by triggering localized actin assembly [8–10]. While this ability of EHEC leads to colonization of the large intestine, production of prophage-encoded Shiga toxin (Stx) promotes intestinal damage resulting in hemorrhagic colitis [11–17]. Shiga toxin may further translocate across the colonic epithelium into the bloodstream, leading to systemic disease. Distal tissue sites, including the kidney, express high levels of the Shiga toxin-binding globotriosylceramide (Gb3) receptor, potentially leading to HUS [14, 15, 18–21].

Genes encoding EHEC Shiga toxin are typically encoded in the late gene transcription region of integrated lambdoid prophages [22, 23] and their expression is thus predicted to be temporally controlled by phage regulons [24–27]. Early studies showed that high levels of Stx production and release from the bacterium *in vitro* required prophage induction, i.e., the mechanism by which quiescent prophages of lysogenic bacteria are induced to replicate intracellularly and released as phage particles by host cell lysis [27, 28]. Lambdoid phage inducers are most commonly agents that damage DNA or interfere with DNA synthesis, such as ultraviolet light or mitomycin C. These inducing stimuli trigger activation of the bacterial RecA protein, ultimately leading to the cleavage of the prophage major repressor protein, CI, allowing expression of phage early and middle genes. Late gene transcription, which requires the Q antiterminator, results in the expression of many virion structural genes and of endolytic functions S and R, which lyse the bacterium and release progeny phage [29]. Other signaling pathways involving quorum sensing or stress responses have also been implicated in lysogenic induction [30, 31].

Unfortunately, antibiotics commonly used to treat diarrheal diseases in children and adults are known to induce the SOS response. Trimethoprim-sulfamethoxazole and ciprofloxacin have been shown to enhance Stx production *in vitro* [32–34], and antibiotic treatment of EHEC-infected individuals is associated with an increased risk of HUS [35]. Hence, antibiotics are contraindicated for EHEC infection and current treatment is limited to supportive measures [36].

A more detailed understanding of the role of prophage induction and Stx production and disease has been pursued in animal models of EHEC infection. Although some strains of conventional mice can be transiently colonized by EHEC, colonization is not robust and typically diminishes over the course of a week [13, 37], necessitating use of streptomycin-treated [16] or germ-free mice [38, 39] to investigate disease manifestations that require efficient, longer-term intestinal colonization. In streptomycin-treated mice colonized with EHEC, administration of ciprofloxacin, a known SOS inducer, induces the Stx prophage lytic cycle, leading to increased Stx production in mouse intestines and to Stx-mediated lethality [40]. Conversely, an EHEC strain encoding a mutant CI repressor incapable of inactivation by the SOS response was also incapable of causing disease in germ-free mice [41].

A potential limitation of the antibiotic-treated or germ-free mouse infection models is the disruption or absence, respectively, of microbiota, with concomitant alterations in immune and physiological function [42]. For example, a laboratory-adapted *E*. *coli* strain that lacks the colonization factors of commensal or pathogenic *E*. *coli* is capable of stably colonizing streptomycin-treated mice [43], and, when overproducing Stx2, is capable of causing lethal infection in antibiotic-treated mice [17]. Further, as up to 10% of human gut commensal *E*. *coli* were found to be susceptible to lysogenic infection by Stx phages *in vitro* [44], it has been postulated that commensals may play an amplifying role in EHEC disease by fostering successive rounds of lytic phage growth [44–47]. Finally, gut microbiota may also directly influence expression of *stx* genes. For example, whereas a genetic sensor of phage induction suggests that the luminal environment of the germ-free mouse intestine harbors a prophage-inducing stimulus [41], several commensal bacteria have been shown to inhibit prophage induction and/or Stx production *in vitro* [48–50]. Alternatively, colicinogenic bacteria produce DNAse colicins that may trigger the SOS response, increasing Stx production [51].

Our laboratory previously developed a murine model for EHEC using the murine AE pathogen *C*. *rodentium* [52, 53], which efficiently colonizes conventionally raised mice and allows the study of infection in mice with intact microbiota. The infecting *C*. *rodentium* is lysogenized with *E*. *coli* Stx2-producing phage Φ1720a-02 [52, 54] encoding Stx variant Stx2_*dact*_ (Stx2d activatable), which is particularly potent in mice [55, 56]. Infection of C57BL/6 mice with *C*. *rodentium*(Φ1720a-02), (herein referred to as *C*. *rodentium*(Φ*stx*_*2dact*_)), produces many of the features of human EHEC infection, including colitis, renal damage, weight loss, and potential lethality, in an Stx_*2dact*_-dependent manner [52].

In the current study, we address phage, bacterial, and host factors that lead to lethal EHEC infection. We found that *C*. *rodentium*(Φ*stx*_*2dact*_) strains lacking RecA, which is required for induction of an SOS response, or phage *Q* protein, which is required for efficient transcription of the late phage genes, did not produce high levels of Stx *in vitro* or cause lethal disease in mice. In contrast, mutants defective in prophage excision, phage assembly, or phage-induced bacterial lysis retained the ability to both produce Stx_*2*dact_ upon prophage induction *in vitro* and to cause lethal disease. Excised phage genomes, potentially undergoing DNA replication leading to phage production or representing packaged phage, were detected, albeit at low levels, in fecal samples of mice infected with wild type *C*. *rodentium*(Φ*stx*_*2dact*_), but not in mice infected with excision-defective *C*. *rodentium*(Φ*stx*_*2dact*_). Thus, in a microbiome-replete EHEC infection model, lytic induction of Stx-encoding prophage, but not actual production of viable phage particles, is essential for Stx production and lethal disease.

## Results

### Gene map and features of Φ*stx*_*2dact*_ prophage

Lambdoid phage Φ1720a-02 was originally isolated from EC1720a-02, a STEC strain found in packaged ground beef [54]. Our novel *C*. *rodentium*-mediated mouse model of EHEC infection encompasses *C*. *rodentium* DBS100 (also known as *C*. *rodentium* strain ICC 168 (GenBank accession number NC_013716.1)), lysogenized with phage Φ1720a-02 marked with a chloramphenicol (*cam*)-resistance cassette inserted into the phage *Rz* gene, creating strain DBS770 [52, 53]. A second lysogen, DBS771, was lysogenized with the same phage but with an additional kanamycin (*kan*)-resistance cassette inserted into and inactivating the prophage *stx2A* gene. For simplicity, strains DBS770 and DBS771 will herein be referred to as *C*. *rodentium* (Φ*stx*_*2dact*_), and *C*. *rodentium*(ΦΔ*stx*_*2dact*_::*kan*^*R*^), respectively (Table 1).

**Table 1:** Bacterial strains and plasmids.

*Strain*	Description	Reference
*C*. *rodentium* wild type	Strain DBS100 (also known as ICC 168).	[57, 58]
*C*. *rodentium* (Φ*stx*_*2dact*_)	DBS770, i.e., DBS100 (Φ1720a-02 Δ*Rz*::*cat*), chloramphenicol^R^	[59] and GenBank accession number KF030445
*C*. *rodentium* (Φ*stx*_*2dact*_::*kan*^*R*^)	DBS771, i.e., DBS770 with a kanamycin resistance cassette inserted into the *stx2A* gene, chloramphenicol and kanamycin resistant	[59]
*C*. *rodentium* (Φ*stx*_*2dact*_ *Δint*)	DBS770 deleted for prophage *int* gene	This study
*C*. *rodentium* (Φ*stx*_*2dact*_ *ΔSR*)	DBS770 deletion for prophage *SR* genes	This study
*C*. *rodentium* (Φ*stx*_*2dact*_ *ΔB*)	DBS770 deleted for prophage *B* gene	This study
*C*. *rodentium* (Φ*stx*_*2dact*_*ΔQ*)	DBS770 with deleted for prophage *Q* gene	This study
*C*. *rodentium ΔrecA* (Φ*stx*_*2dact*_)	DBS770 deleted for host *recA* gene	This study
*C*. *rodentium ΔrpoS* (Φ*stx*_*2dact*_)	DBS770 deleted for host *rpoS* gene	This study
*C*. *rodentium ΔqseC (Φstx*_*2dact*_*)*	DBS770 deleted for host *qseC* gene	This study
*C*. *rodentium ΔqseF (Φstx*_*2dact*_*)*	DBS770 deleted for host *qseF* gene	This study
*E*. *coli* K12 DH5α	*fhuA2 lac(del)U169 phoA* *glnV44 Φ80' lacZ(del)M15* *gyrA96 recA1 relA1 endA1* *thi-1 hsdR17*	[60]
**Plasmids**	**Description**	**Reference**
pKD46	Phage Lambda-*red* recombinase, *bla*	[61]
pTOPO-Q	pCR4 TOPO vector encoding *Q* gene and 100 bp region upstream	This study

To identify phage genes critical for lethal mouse infection, we sought to inactivate specific prophage genes and then assess their resulting phenotypes in the *C*. *rodentium* mouse model. As a first step, we sequenced the parental strain DBS100 and the genomes of *C*. *rodentium* (Φ*stx*_*2dact*_) and *C*. *rodentium* (ΦΔ*stx*_*2dact*_::*kan*^*R*^), revealing that the three genomes were identical except for prophage sequences present in *C*. *rodentium* (Φ*stx*_*2dact*_) and *C*. *rodentium* (ΦΔ*stx*_*2dact*_::*kan*^*R*^) (see Materials and Methods).

**Fig 1:**
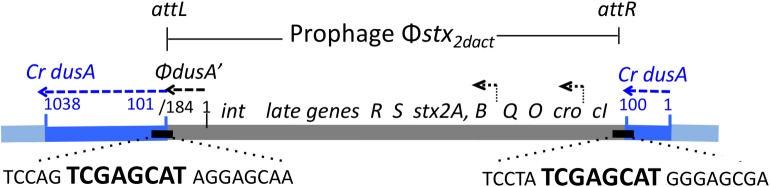
Prophage Φ*stx*_*2dact*_ in *C*. *rodentium* (Φ*stx*_*2dact*_). The Φ*stx*_*2dact*_ prophage (gray), flanked by *attL* and *attR* upon insertion into *C*. *rodentium dusA* sequence (blue, “*Cr dusA*”), was determined by whole genome sequencing of *C*. *rodentium*(ΦΔ*stx*_*2dact*_::*kan*^*R*^). The 3’ end of the prophage (nucleotides 1–184) encodes the N-terminal 61 residues of “*ΦdusA*,*’*” in the same reading frame as the 3’ end (nucleotides 101–1038) of the *C*. *rodentium dusA* gene (“*Cr dusA*”). Bent arrows indicate direction of transcription of *Q*, *stx*, and phage late genes. Depicted are *attL* and *attR* sequence motifs, characteristic of other prophages inserted within the host *dusA* gene ([63]). Within this sequence, a seven-base “core” sequence (bolded), perfectly conserved in *attL* and *attR*, as well as in the Φ*stx*_*2dact*_ *attP* sequence shown here and in the parental *attB* sequence in *C*. *rodentium dusA* (TCCAG**TCGAGCAT**GGGAGC), is the cross-over site for phage integration and excision.

We then annotated the entire Φ*stx*_*2dact*_ prophage (GenBank accession number KF030445.1; Figs 1 and S1). As is typical of Stx phages, the sequence revealed a lambdoid phage with a mosaic gene organization that does not precisely match that of phage λ, but is nevertheless somewhat syntenic with other lambdoid phages [62], (S2 Fig). Further, although lysogenized independently, *C*. *rodentium*(Φ*stx*_*2dact*_) and *C*. *rodentium*(ΦΔ*stx*_*2dact*_::*kan*^*R*^) prophages were integrated at the same location, i.e. 100 bp into the coding sequence of *dusA* (encoding tRNA-dihydroxyuridine synthase A). A recent study revealed that known integrase genes, at least half of which belong to prophages, were found adjacent to the host *dusA* gene in over 200 bacterial species [63]. Furthermore, a 21 base pair motif found at the prophage-host DNA junctions in many bacteria was present at the prophage junctions, *attL* and *attR*, of *C*. *rodentium*(Φ*stx*_*2dact*_) and *C*. *rodentium*(ΦΔ*stx*_*2dact*_::*kan*^*R*^), as well as at the presumed *attB* phage insertion site in the parental *C*. *rodentium dusA* gene (Fig 1). A seven-base segment within this 21-base sequence is completely conserved between *attL*, *attR*, and *attB* and likely represents the ‘core’ recombination site for integration or excision (Fig 1, bolded sequence; [64]). Note that, although the Φ*stx*_*2dact*_ and ΦΔ*stx*_*2dact*_::*kan*^*R*^ prophages interrupt the *dusA* gene, they encode a 184 bp ORF (designated “Φ*dusA’”* in Fig 1) that is in frame with the 3’ 937 nucleotides (positions 101 to 1038) of *dusA*

**Fig 2:**
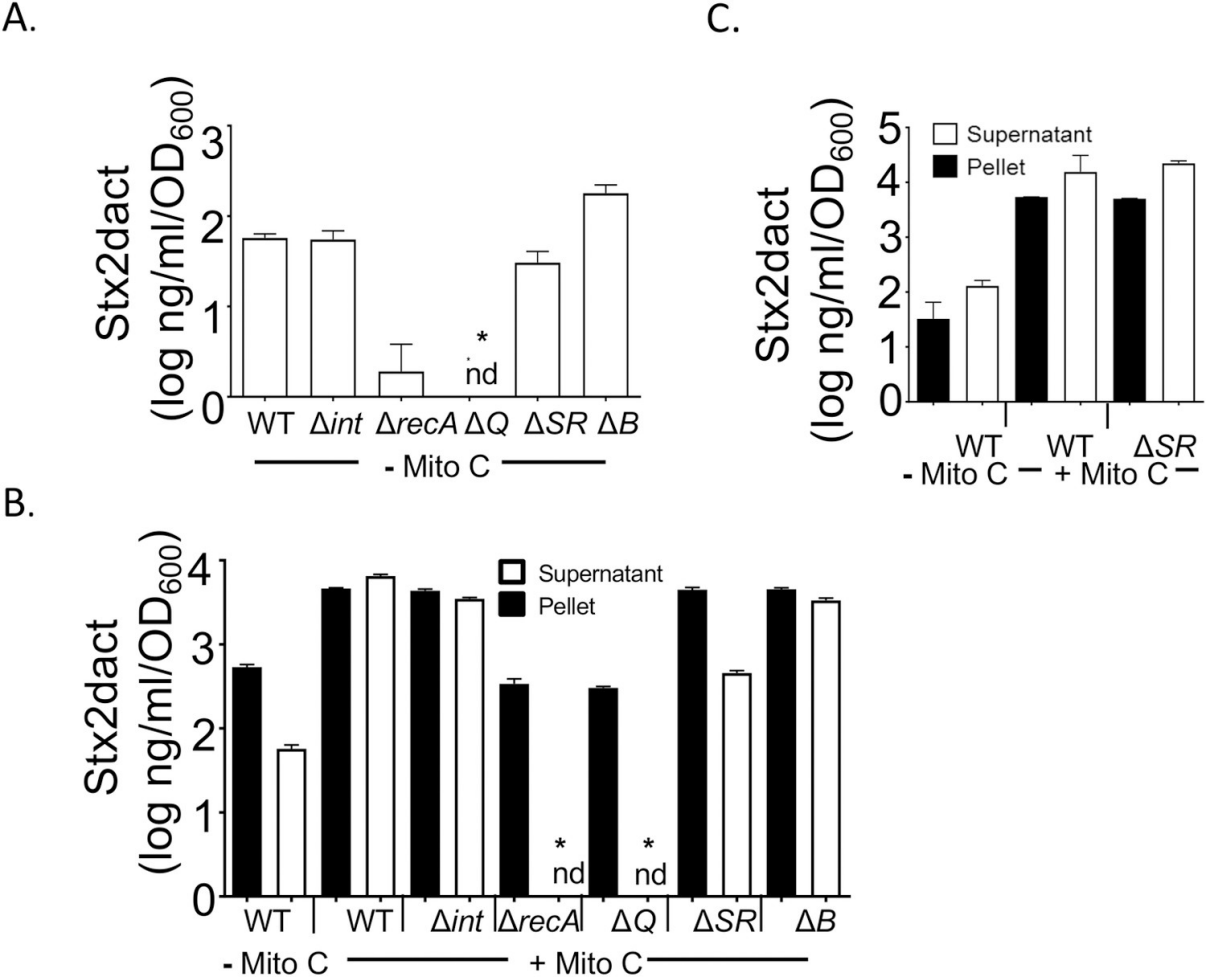
SOS responsiveness and lytic induction-dependent transcription of *stx* genes are required for wild type basal and induced levels of Stx2 production *in vitro*. **A.** The indicated lysogens were grown in the absence of mitomycin C until t = 4h, i.e., four hours after attaining approximately mid-log phase (which was designated as t = 0h; “- Mito C”), and culture supernatants were subjected to capture ELISA to determine the basal level of Stx2 production (see Materials and Methods). Quantities are expressed relative to the specific OD_600_ at t = 0h. nd: not detected. **B.** The indicated lysogens were grown to mid-log phase (t = 0h) and cultured for four more hours (t = 4h) either in the absence (“- Mito C”) or presence of 0.25 μg/ml mitomycin C (“+ Mito C”). Pellets (filled bars) or supernatants (open bars) were subjected to capture ELISA to determine the level of Stx2 production. Quantities are expressed relative to the specific OD_600_ at t = 0h. nd: not detected. **C.** Wild type *C*. *rodentium*(Φ*stx*_*2dact*_) and *C*. *rodentium*(Φ*stx*_*2dact*_ Δ*RS*)) were grown to mid-log phase (designated as t = 0h) and cultured for 16 more hours (t = 16h) either in the absence (“- Mito C”) or presence of 0.25 μg/ml mitomycin C (“+ Mito C”). Pellets (filled bars) or supernatants (open bars) were subjected to capture ELISA to determine the level of Stx_*2dact*_ production. Quantities are expressed relative to the specific OD_600_ at t = 0h. For all panels, results are averages ± SEM of triplicate samples, and are a representative of at least two experiments involving independently derived mutants. Asterisks (*) indicate Stx level significantly (*p* <0.05) different from wild type *C*. *rodentium* (Φstx_*2dact*_) calculated using Kruskal–Wallis one-way analysis of variance followed by Dunn's nonparametric comparison.

A prior analysis of the host *C*. *rodentium* DBS100 genome sequence revealed the presence of 10 additional partial and intact prophages distributed around the genome [65], although it is not known if any of these prophages can give rise to intact phage. Sequence analysis showed only 2 regions of homology between Φ*stx*_*2dact*_ and these prophages (S3 Fig): one resident prophage encoded 70% homology to a region encoding Φ*stx*_*2dact*_ Cro, CI repressor, and a hypothetical protein, and a second resident prophage showed 79% homology to another Φ*stx*_*2dact*_ gene encoding a hypothetical protein.

### Survey of prophage integration (*att*) sites during murine infection reveals Φ*stx*_*2dact*_ prophage excision from *C*. *rodentium*(Φ*stx*_*2dact*_), but no secondary lysogeny of commensal bacteria

Although Φ*stx*_*2dact*_ harbors a *cat* insertion in the *Rz* gene, a gene that contributes to phage λ lysis under some conditions [2], prophage induction with mitomycin C resulted in lysis of *C*. *rodentium*(Φ*stx*_*2dact*_) (S4 Fig), suggestive of lytic phage induction. Nevertheless, pilot experiments revealed that Φ*stx*_*2dact*_ plaques were not detectable on any of numerous *E*. *coli* K12 and other indicator strains (Materials and Methods). This finding is not unusual for Stx-producing phages [66–68]. To more rigorously test whether this phage can infect *E*. *coli* K12, we selected for *E*. *coli* K12 lysogens by infecting *E*. *coli* K12 strain DH5α with supernatants of mitomycin C -induced cultures of *C*. *rodentium* (Φ*stx*_*2dact*_), then selecting for kanamycin-resistant clones. These clones were verified as lysogens by PCR-detection of phage genes (S5A Fig). DH5α lacks RecA and thus cannot undergo an SOS response to trigger prophage induction. However, when the RecA-producing plasmid pER271 was introduced to the DH5α lysogens, they were more sensitive to UV light than non-lysogens containing the same plasmid (S5B Fig), consistent with lysogenic induction. Hence, Φ*stx*_*2dact*_ is a functional phage that is capable of infecting bacteria, including *E*. *coli* K12.

In the course of EHEC infection of streptomycin-treated mice, Stx phage can be induced by antibiotic treatment to lysogenize other *E coli* strains in the intraluminal environment [40] [69]. It has been postulated that successive cycles of infection of non-pathogenic commensal *E*. *coli* could amplify Stx production and exacerbate disease [38, 44, 45, 47]. We first addressed this question by testing whether lysogeny of commensal bacteria by phage Φ*stx*_*2dact*_ was detectable following oral *C*. *rodentium*(Φ*stx*_*2dact*_) infection of mice. Mice orally gavaged with *C*. *rodentium*(Φ*stx*_*2dact*_) normally exhibit weight loss and lethal disease [52], typically succumbing to disease after day 7 post-infection. DNA was extracted from fecal samples of a group of five mice at days 1 and 6 post-infection. The DNA samples were used as a template to generate a library of sequences encompassing the sequence downstream of *attL* (specifically, spanning the region from the phage *int* gene, through Φ *dusA* and into the adjacent host sequence; see Fig 1). This strategy is a modification of that used for *Tn*-seq library analysis ([70], Materials and Methods).

Although we were unable to obtain detectable amplified DNA from fecal samples produced on day 1 post-infection, consistent with the low titer of *C*. *rodentium*(Φ*stx*_*2dact*_) in the stool at this early time point, the day 6 post-infection sample yielded a DNA library, which was subjected to massively parallel sequencing to identify the origin of the host DNA into which the prophage was integrated. Of 17,142,098 readable sequences generated, 99.56% showed homology to *C*. *rodentium* (Φ*stx*_*2dact*_), i.e. included *C*. *rodentium* (Φ*stx*_*2dact*_) *attL* and the adjacent *C*. *rodentium dusA* gene sequence, indicating prophage integration into the original *C*. *rodentium* strain (Table 2; see Materials and Methods). For the remaining 0.44% of sequences, the *C*. *rodentium dusA* sequences adjacent to the *attL* core sequence were replaced by phage-specific *attR* sequences, thus regenerating *attP*. These latter sequences likely reflect excised circular phage genomes generated following induction of the *C*. *rodentium*(Φ*stx*_*2dact*_) lysogen. Thus, *C*. *rodentium*(Φ*stx*_*2dact*_) undergoes lytic induction in the murine host, consistent with previous findings of EHEC infection in streptomycin-treated mice. Furthermore, no integration of the Φ*stx*_*2dact*_ prophage into either a different site in *C*. *rodentium*, or into a different bacterial host was observed, leading to the conclusion that lysogeny of intestinal bacteria by Φ*stx*_*2dact*_ is not a common event in this model.

**Table 2:** Comprehensive survey of prophage attachment (integration) sites reveals prophage excision but not secondary lysogeny of commensal bacteria during murine infection by *C. rodentium* (Φstx2dact).

Sequence identity	Number	Percent of total
*C*. *rodentium*(Φ*stx*_*2dact*_) *attL*	17,066,136	99.56
Φ*stx*_*2dact*_ *attP* (replicative form)	75,962	0.44
**Total sequences**^1^	17,142,098	100.00

^1^Of a total of 17,868,095 sequences, 725,997 were of poor quality, resulting in a total of 17,142,098 readable sequences.

### *C*. *rodentium* RecA and Φ*stx*_*2dact*_ proteins integrase, Q, endolysins, and portal protein are required for efficient phage production and release *in vitro*

Prophage induction of lambdoid phages is often initiated by DNA damage, in which SOS pathway activation leads to RecA-promoted autocleavage of *C*I repressor, followed by transcription of early genes from the from P_L_ and P_R_ promoters. Subsequent temporally programmed transcription of the prophage genome results in the production of delayed early (middle) proteins such as Int (integrase), essential for prophage integration and excision, and antiterminator protein Q. Production of Q in turn mediates the transcription of late genes, including portal protein gene *B*, responsible for translocation of phage DNA into the virion protein capsid, and lysis genes *S* and *R*, encoding endolysins that disrupt the bacterial plasma membrane causing release of intact phage progeny (for a review, see Gottesman and Weisberg [71]). Late genes in EHEC phages also encompass *stx*.

To uncover the roles of specific phage and bacterial functions in EHEC disease, we used lambda *red* recombination (Materials and Methods) to construct *C*. *rodentium*(Φ*stx*_*2dact*_) strains defective for prophage genes *SR*, *int*, *B*, or *Q*, or the host gene *recA*, which is well documented to be central to the SOS response and lytic induction. In addition, we inactivated three other genes that have been implicated as having more subtle roles in the lytic induction of Shiga toxin-encoding phage [30, 31]: *rpoS*, which controls the bacterial stress response, and *qseC* and *qseF*, which control quorum sensing pathways (Materials and Methods, Table 1). The production of Shiga toxin phage has been shown to be influenced by growth medium [66], but none of the mutants displayed a growth defect upon *in vitro* culture in LB or DMEM medium (S6 Fig).

We then tested *C*. *rodentium*(Φ*stx*_*2dact*_) and several of the mutant derivatives predicted to have dramatic effects on phage production for the ability to generate Φ*stx*_*2dact*_ following SOS induction. Given that Φ*stx*_*2dact*_ was found to not form plaques on indicator strains tested, we instead utilized qPCR to quantify phage [72–74]. Specifically, we employed primers flanking the phage *att*P site to distinguish integrated and excised phage DNA, as only the latter will have reconstituted the *attP* site [71]. This technique detects both unpackaged phage genomes and those packaged in phage capsids, as in our initial experiments lysates were not treated with DNase prior to qPCR enumeration. Note that protease digestion of the capsid prior to qPCR quantitation was also eliminated, as capsid undergoes melting during the high heating steps of the PCR procedure [75]

Supernatants of mid-logarithmic phase (t = 0h) LB cultures contained 1.3×10^9^–3.8×10^9^ *attP* copies (phage genomes) per ml (Table 3 legend), compared to approximately 10^8^ viable bacteria per ml, indicating significant spontaneous prophage induction during the period leading to mid-log growth. After four additional hours (t = 4h), supernatant phage concentration increased 3.2-fold relative to t = 0h, consistent with continued spontaneous prophage induction (Table 3, “Relative *attP* production”, “- Mito C”). Prophage induction of the wild type lysogen with the SOS inducer mitomycin C led to a 234-fold increase in relative *attP* production (Table 3, “+ Mito C”), a 73-fold increase above baseline levels. As predicted [76, 77], the generation of circular phage genomes required Int recombinase, as at all time points tested, *attP* copies were below the level of detection of 1× 10^4^/ml in uninduced or mitomycin C-induced cultures of the *C*. *rodentium*(Φ*stx*_*2dact*_Δ*int*) mutant (Table 3).

**Table 3:** *C*. *rodentium* RecA and Φ*stx*_*2dact*_ proteins integrase, Q, endolysins, and portal protein are required for efficient phage production and release *in vitro*.

Strain	Function deleted	Relative a*ttP* (phage) production		
		- Mito C^a^	+ Mito C^b^	+Mito C + DNAse^c^
WT	None (WT)	3.2 (±0.01)	234.6 (±24.4)	162.5 (±1.1)
Δ*int*	Phage integrase	Not detected	Not detected	Not determined
Δ*recA*	Host RecA	0.5 (±0.6)*	29.4 (±11.4)*	Not determined
Δ*Q*	Phage late gene transcription anti-terminator	0.5 (±0.3)*	6.0 (±0.3)*	Not determined
Δ*SR*	Phage endolysin	0.6 (±0.2)*	6.3 (±2.0)*	Not determined
Δ*B*	Phage portal protein	4.1 (±0.08)	208.7 (±17.2)	9.2 (±1.3)*

^a^Supernatants from mid-log (t = 0h) cultures or parallel cultures grown for an additional 4 hours (t = 4h) were analyzed for *attP* copies by qPCR. Shown are average values of t = 4h/t = 0h (+/- SEM) for each lysogen, derived from the values of three different dilutions of each supernatant (see Materials and Methods). For all lysogens except *C*. *rodentium*(Φ*stx*_*2dact*_*Δint*), absolute numbers of *attP* molecules at t = 0h ranged from 1.3×10^9^ to 3.8×10^9^/ml. For *C*. *rodentium*(Φ*stx*_*2dact*_*Δint*), *attP* copies were below the limit of detection, i.e., <1× 10^4^/ml.

^b^Supernatants from mid-log (t = 0h) cultures, and parallel cultures subsequently exposed to 0.25 μg/ml mitomycin C for 4 hours (t = 4h) were analyzed for *attP* copies by qPCR and the ratios of the two values determined as above. For *C*. *rodentium*(Φ*stx*_*2dact*_ *int*), *attP* copies were below the limit of detection, i.e., <1× 10^4^/ml.

^c^Supernatants from mid-log (t = 0h) cultures or parallel cultures subsequently exposed to 0.25 μg/ml mitomycin C for 4 hours (t = 4h) were analyzed for *attP* copies by qPCR after treatment with DNase (1 hr, according to manufacturer’s instructions), to remove unpackaged DNA. The ratios of the two values were determined as above. Note that DNase treatment longer than 1 hr did not significantly alter the results.

*indicates statistical significance (p<0.05) compared to identically treated WT, calculated by one-way Anova.

Host and phage functions contributed to the amount of phage production. In the absence of inducer (Table 3, “- MitoC”), the concentration of *attP* copies in culture supernatants of *C*. *rodentium*Δ*recA*(Φ*stx*_*2dact*_), predicted to be defective for SOS induction, did not increase between t = 0h and t = 4h, with an average relative *attP* production of 0.5. Lysogens deficient in the antiterminator Q, required for late gene transcription, or deficient in the S and R endolysins, which promote the efficient release of phage from infected bacteria, were also deficient in relative *attP* production in the absence of inducer (Table 3). Finally, *C*. *rodentium*(Φ*stx*_*2dact*_Δ*B*), predicted to replicate but not package phage genomes, showed no defect in the production of *attP* copies in the culture supernatant in the absence of inducer, with relative phage production ratio of 4.1. However, as described below, DNAse sensitivity assays suggested that these *attP* sequences are likely not packaged into phage particles.

The mutants defective in baseline phage production were similarly defective in the titer of *attP* copies after induction with mitomycin C (Table 3, “+ Mito C”). Induction of *C*. *rodentium*Δ*recA* (Φ*stx*_*2dact*_) resulted in an increase in *attP* production, consistent with low levels of phage production by RecA-deficient λ lysogens of *E*. *coli following* induction [78], but the relative *att*P value of 29 was eight-fold lower than wild type. *C*. *rodentium*(Φ*stx*_*2dact*_Δ*Q*) and *C*. *rodentium*(Φ*stx*_*2dact*_Δ*SR*) each also demonstrated dramatically diminished *attP* copies in mitomycin C-induced culture supernatants, with relative *attP* production of approximately 6. The small increase in *att*P levels for each of these mutants upon induction is consistent with readthrough of early transcription of Q-deficient λ mutants [79] and low level bacterial lysis in the absence of phage-encoded endolysins, respectively.

Finally, *C*. *rodentium* (Φ*stx*_*2dact*_Δ*B*), generated wild type levels of phage genome copies, with a 209-fold increase in relative *attP* production. However, DNAse treatment of supernatants diminished this value more than 23-fold, whereas parallel treatment diminished the relative *attP* production by wild type *C*. *rodentium*(Φ*stx*_*2dact*_) less than 1.5-fold (Table 3, “+Mito C + DNAse”), consistent with a defect in packaging of Φ*stx*_*2dact*_ genomes in the absence of the B portal protein.

### Proteins required for the SOS response and/or late gene transcription are essential for Stx_*2dact*_ production

To determine which host or phage functions are required for production of Stx_*2dact*_ *in vitro*, we measured Stx_*2dact*_ in culture supernatants by ELISA [53]. To quantitate non-induced levels of toxin, and to provide ample time for toxin to accumulate, we grew triplicate cultures of the *C*. *rodentium*(Φ*stx*_*2dact*_) or the mutant derivatives described above for four hours (t = 4h) beyond mid-log phase (defined as t = 0h). Stx_*2dact*_ was present in the culture supernatants of wild type *C*. *rodentium*(Φ*stx*_*2dact*_) at approximately 50 ng/ml/OD_600_ unit, consistent with previous measurements [53] (Fig 2A, “WT”). Prophage excision and phage production were not required for this basal level of Stx_*2dact*_: culture supernatants of *C*. *rodentium*(Φ*stx*_*2dact*_Δ*int*), which did not harbor detectable phage (Table 3), contained equivalent amounts of toxin (Fig 2A, “Δ*int*”). Uninduced culture supertants of *C*. *rodentium*(Φ*stx*_*2dact*_Δ*SR*) contained levels of Stx_*2dact*_ two-fold lower than (and statistically indistinguishable from) wild type, consistent with the moderately (5-fold) lower levels of phage found in cultures of wild type *C*. *rodentium* (*Φstx*_*2dact*_) (Table 3, “- MitoC”). Supernatants of *C*. *rodentium*(Φ*stx*_*2dact*_Δ*B*), which contained *attP* DNA but relatively few packaged phage (Table 3), also produced levels of Stx_*2dact*_ statistically indistinguishable from wild type. Finally, in contrast, *C*. *rodentium*Δ*recA* (Φ*stx*_*2dact*_), which is unable to mount an SOS response, and *C*. *rodentium*(Φ*stx*_*2dact*_Δ*Q*), which cannot transcribe phage late genes, including *stx*_2*dactA*_ and *stx*_2*dactB*_, were defective for basal levels of Stx_*2dact*_ production (Fig 2A, “Δ*recA*”, “Δ*Q*”).

To test whether the defect in Stx_*2dact*_ production was due to the lesion in the Q gene, we complemented *C*. *rodentium*(Φ*stx*_*2dact*_Δ*Q*) with plasmid pTOPO-Q, the wild type Q gene (Table 1). The complemented strain indeed increased Stx_*2dact*_ production 286-fold (S7 Fig, “ΔQ + pTOPO-Q”). Nevertheless, this level of Stx_*2dact*_ was 12-fold lower than that produced by the wild type *C*. *rodentium* (Φ*stx*_*2dact*_) strain, a defect that is likely due to unregulated Q production in trans [80] because we found that pTOPO-Q similarly diminished Stx production by the WT strain (S7 Fig, “WT + pTOPO-Q”). The exquisite developmental control of gene expression during the lysogenic and lytic cycle is a hallmark of lambdoid phages [23], making complementation of many of phage mutants technically challenging [80].Hence, to minimize the risk that phenotypes observed were due to off-target lesions, we isolated two independent clones of each mutant and tested both clones for each of the phenotypes observed throughout this study.

We also assessed toxin production by wild type *C*. *rodentium*(Φ*stx*_*2dact*_) and mutant derivatives after 4h of mitomycin C induction. Given that mitomycin C-induced Φ*stx*_*2dact*_ functions may be involved in the release of toxin from the bacterial host [27], we assessed toxin in cell pellets and in culture supernatants separately. As previously observed [52], mitomycin C induction resulted in a more than 100-fold increase of Stx_*2dact*_ in culture supernatants (Fig 2B, “WT"). A nearly equivalent amount of toxin remained associated with the bacterial cell pellet, suggesting that under these conditions, a significant fraction of bacteria remained unlysed. Culture supernatants or cell pellets of the *C*. *rodentium*Δ*rpoS* (Φ*stx*_*2dact*_) mutant predicted to be defective in the bacterial stress response, or the *C*. *rodentium*Δ*qseC*(Φ*stx*_*2dact*_) and *C*. *rodentium*Δ*qseF*(Φ*stx*_*2dact*_) mutants defective for quorum sensing, showed wild type levels of Stx_*2dact*_ (S8 Fig), indicating that neither the bacterial stress response nor the QseC- or QseF-mediated quorum responses were required for toxin production. Culture supernatants of *C*. *rodentium*(Φ*stx*_*2dact*_Δ*int*) and *C*. *rodentium*(Φ*stx*_*2dact*_Δ*B*), which showed no defect in basal levels of toxin production (Fig 2A), also contained amounts of cell-associated toxin and supernatant-associated Stx_*2dact*_ indistinguishable from wild type (Fig 2B,”Δ*int*” and” Δ*B*”), despite the lack of prophage excision and/or phage production in these mutant strains. The Δ*SR* lysogen, defective for phage endolytic functions, produced wild type levels of cell-associated Stx_*2dact*_ at 4 h post-induction, but supernatant-associated toxin was approximately ten-fold lower than wild type levels (Fig 2B, “Δ*SR*”). This difference is consistent with a defect in bacterial lysis and Stx_*2dact*_ release, but did not reach statistical significance. In addition, by 16 h post-induction of the *ΔSR* lysogen, Stx_*2dact*_ was detected in supernatants at levels similar to that of the WT strain (Fig 2C), suggesting that any defect in R and S proteins results in a delay rather than an absolute block in toxin release. Finally, however, deficiency in the RecA or Q proteins was associated with a near-complete absence of Stx_*2dact*_ in cell supernatants (Fig 2B,”Δ*recA*” and” Δ*Q*”), reinforcing the notion that these proteins, which are required for the SOS response and/or transcription of the *stx2*_*dact*_ genes ([81] [71]), are essential for large amounts of Stx_*2dact*_ production.

### *C*. *rodentium*(Φ*stx*_*2dact*_) undergoes lytic induction during murine infection

**Fig 3:**
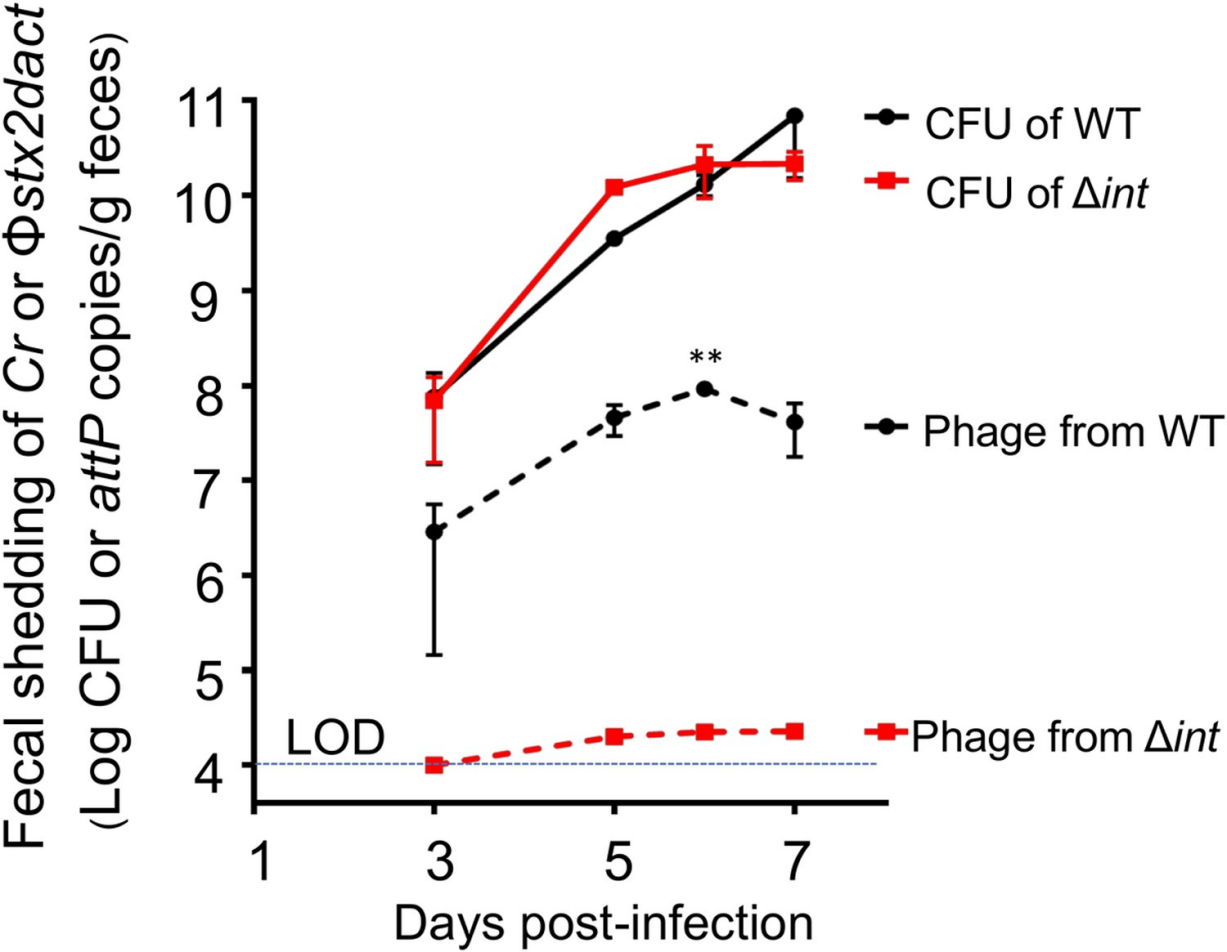
*C*. *rodentium*(Φ*stx*_*2dact*_) undergoes lytic induction during murine infection. Eight-week old female C57BL/6 mice were infected by oral gavage with *C*. *rodentium*(Φ*stx*_*2dact*_) or *C*. *rodentium*(Φ*stx*_*2dact*_ Δ*int*). At the indicated time points, *attP* copies, reflecting excised prophages, and viable bacteria were determined by qPCR or plating for CFU, respectively (see Materials and Methods). Shown are averages ± SEM of 5 mice per group of a representative of two experiments. Level of detection of *attP* was 1 x 10^4^ copies/g feces. Asterisks (**) indicate significance differences (*p* <0.01) between the WT and *C*. *rodentium* (Φstx_*2dact*_ Δ*int*) calculated using 2-way ANOVA followed by Bonferroni post tests.

Stx-encoding prophages undergo lytic induction during EHEC infection of germ-free or antibiotic-treated mice [40, 41, 69], and our comprehensive survey of prophage integration sites in fecal microbiota (Table 2) indicated that *C*. *rodentium*(Φ*stx*_*2dact*_) undergoes some degree of lytic induction during infection of conventional mice. To assess this induction further, we infected conventionally raised C57BL/6 mice with *C*. *rodentium*(Φ*stx*_*2dact*_) by oral gavage and measured fecal shedding of both the infecting strain, by plating for CFU, and Φ*stx*_*2dact*_, by quantitating *attP* (non-integrated phage) copies by qPCR. As previously observed, by day 3 post-infection, *C*. *rodentium*(Φ*stx*_*2dact*_) was detected in the stool at 8 x 10^7^ per gram, and reached 9 x 10^10^ per gram by day 6 post-infection ([82]; Fig 3, “CFU of WT"). Further, murine infection by this strain was indeed associated with lytic induction, as excised phage genomes were detected in stool at all time points (Fig 3, “Phage from WT”).

Interestingly, given the relatively high phage production by induced *C*. *rodentium*(Φ*stx*_*2dact*_) *in vitro*, the amount of phage detected in stool was quite low. At day 3 post-infection, 5 x 10^6^ *attP* copies were detected per gram of stool, a value 16-fold lower than the concentration of viable *C*. *rodentium*(Φ*stx*_*2dact*_) in stool at that time point. By day 6 post-infection, *attP* copies had increased to 5 x 10^7^ per gram of feces, but were approximately 600-fold lower than the fecal bacterial counts. These results indicate that *C*. *rodentium*(Φ*stx*_*2dact*_) thus undergoes lytic induction and growth in this murine model, although not to the degree seen upon induction *in vitro*.

### Lethal disease in mice correlates with the ability to produce Stx2_*dact*_ but not with the ability to produce phage

**Fig 4:**
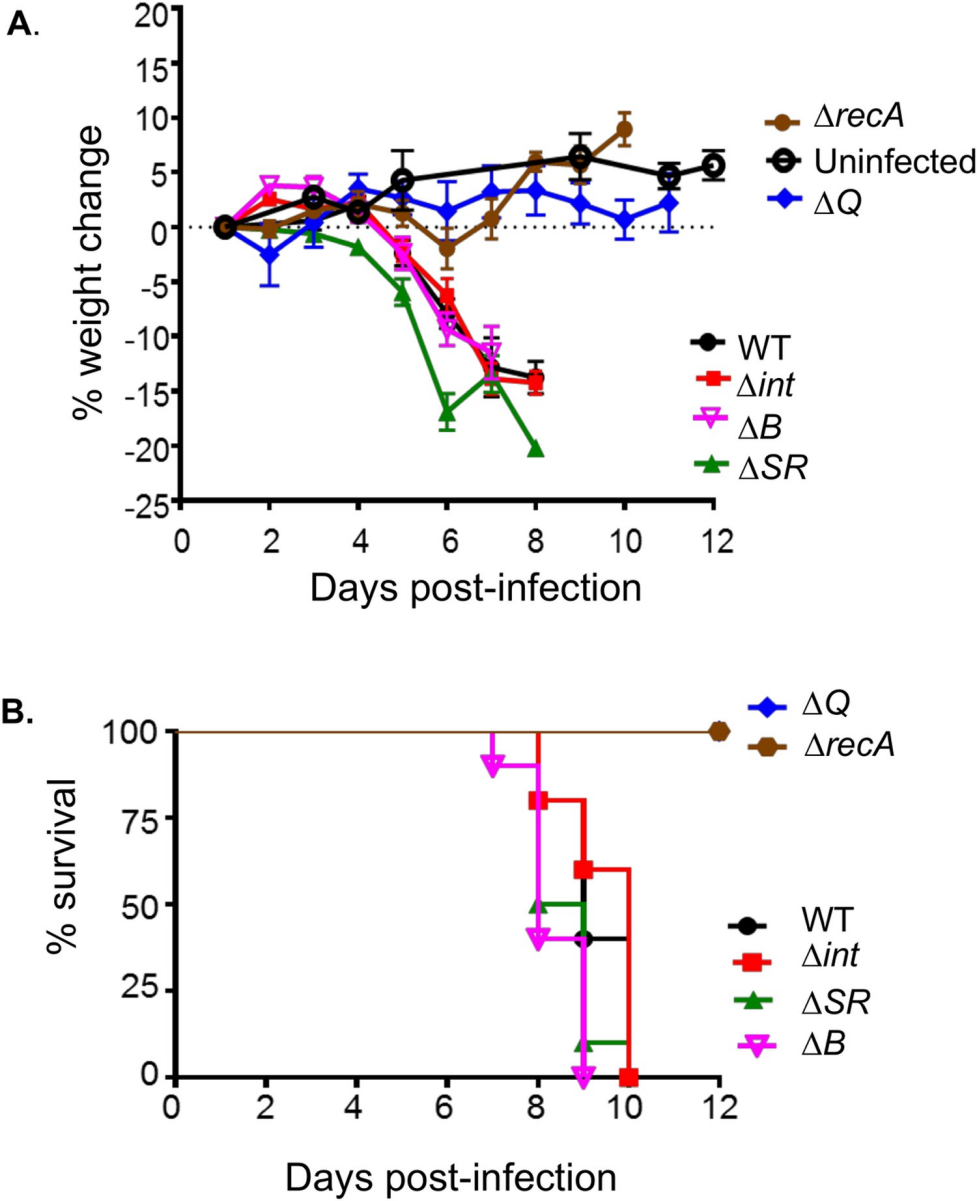
Lethal disease in mice correlates with the ability to produce Stx_*2dact*_ but not with the ability to produce phage. Eight-week old female C57BL/6 mice were infected by oral gavage with the indicated lysogens. **A.** Percentage weight change was determined at indicated post-infection time. Data shown are averages ± SEM of 10 mice per group. Asterisks (*, **) indicate significance (p <0.05, <0.01) determined by 2-way ANOVA followed by Bonferroni post tests. **B.** Percent survival at the indicated post-infection time was monitored in 10 mice per group. Data represent cumulative results of 3 separate experiments.

To test the importance of SOS induction and phage functions on disease in our microbiota-replete model of infection, we infected C57BL/6 mice with *C*. *rodentium*(Φ*stx*_*2dact*_) and mutant derivatives by oral gavage. The wild type and the mutant lysogens colonized mice similarly, although the *ΔB* and *Δint* mutant lysogens appeared to colonize at somewhat higher levels (S9 Fig). *C*. *rodentium* Δ*recA*(Φ*stx*_*2dact*_) and *C*. *rodentium*(Φ*stx*_*2dact*_Δ*Q*), the two mutant lysogens that displayed dramatic defects in basal and mitomycin C-induced levels of Stx_*2dact*_ *in vitro*, were the only ones incapable of causing sickness or death (Fig 4,”Δ*recA*” and”Δ*Q*”), supporting the hypothesis that induction of an SOS response and the subsequent expression of phage late genes, including *stx* genes, are required for Shiga toxin production during infection of a microbiota-replete host.

The RpoS-deficient and QseC-deficient *C*. *rodentium*(Φ*stx*_*2dact*_) mutants that are compromised in bacterial stress and quorum-sensing responses, respectively, retained the ability to cause weight loss and lethality with kinetics that were indistinguishable from that of WT *C*. *rodentium*(Φ*stx*_*2dact*_) (S10 Fig). Thus, although previous results indicated that some quorum sensing mutants display diminished virulence during infection by non-Stx-producing *C*. *rodentium* [83], our results are consistent with the the ability of these strains to produce wild type levels of Stx_*2dact*_ after SOS induction (S8 Fig). In addition, the lack of endolysins that appeared to somewhat delay release of Stx_*2dact*_ into supernatants by *C*. *rodentium* (Φ*stx*_*2dact*_Δ*SR*) was not reflected by any delay in the kinetics of weight loss or lethality in infected mice (Fig 3,”Δ*SR*”), consistent with the ability of this strain to produce wild type levels of Stx_*2dact*_ upon extended culture *in vitro*. Thus, *C*. *rodentium* (Φ*stx*_*2dact*_Δ*SR*) is capable of triggering Stx_*2dact*_–mediated disease in the absence of phage-induced lysis.

Finally, the production of intact phage is not essential to disease in this model. *C*. *rodentium* (Φ*stx*_*2dact-*_Δ*B*), which is unable to generate intact phage *in vitro*, and *C*. *rodentium*(Φ*stx*_*2dact*_Δ*int*), which can neither generate excised phage genomes *in vitro* or *in vivo*, both retained full virulence in this model. We conclude that in this microbiota-replete model of EHEC infection, disease progression correlates exclusively with the ability to produce Stx_*2dact*_, regardless of the lysogen’s ability to amplify the *stx2* genes by phage excision and genome amplification, or by the production of phage that are capable of secondary infection of commensal bacteria.

## Discussion

Commensal organisms have the potential to suppress or enhance phage induction and Stx production. Although a role for induction of *stx*-encoding prophages in the production of Stx and serious disease during animal infection has been well documented in antibiotic-treated and germ-free mice [40, 41, 69], we used a murine model of EHEC infection that features an intact microbiome.

To investigate phage functions required for *C*. *rodentium*(Φ*stx*_*2dact*_) to produce Stx and cause disease in conventional mice, we first characterized prophage genetic structure. Φ*stx*_*2dact*_ prophage was integrated into the *C*. *rodentium dusA* gene, an integration site utilized by prophages in over 200 bacterial species [63]. Although the orientation of the regulatory and late genes within the Φ*stx*_*2dact*_ prophage is noncanonical with respect to *attL* and *attR* (with *int* adjacent to *attL*; Fig 1), this orientation has been previously observed in at least one other lambdoid phage. In addition, Φ*stx*_*2dact*_ genes encoding several key phage proteins were identified by homology, and their inactivation had the predicted effects on phage development and production (Table 3; [77]). For example, antiterminator Q and integrase were required for phage production, as measured by detection of *attP*, and portal protein B was required for packaging of phage DNA into DNAse-resistant virions.

Stx production *in vitro* by the prophage mutants, as well as by a host *recA* mutant, confirmed that prophage induction, i.e., the SOS-dependent process required to initiate a temporal program of phage gene expression that normally leads to phage lytic growth, is essential for high-level Stx2 production *in vitro*. Mitomycin C treatment of *C*. *rodentium*(Φ*stx*_*2dact*_) resulted in a greater than 100-fold increase in Stx_*2dact*_ in culture supernatants, similar to the mitomycin C-mediated increase in Shiga toxin production by EHEC ([41]; Fig 2). Three signaling pathways, mediated by RpoS, QseC, and QseF, previously demonstrated to influence SOS induction of EHEC *in vitro*, had no effect on Stx_*2dact*_ production by *C*. *rodentium* (Φ*stx*_*2dact*_). In contrast, and as expected, RecA, required for mounting an SOS response, was necessary for this enhanced production of Stx_*2dact*_ (Fig 2). It was previously shown that inactivation of the EHEC prophage repressor CI, a key step in the SOS response, is required for the increase in EHEC Stx production upon mitomycin C induction *in vitro* [41].

Despite the previous observation that the increase in phage genome copy number plays the most quantitatively important role in mitomycin C-enhanced Stx1 production by Stx phage H-19B [27], we found that integrase-deficient *C*. *rodentium*(Φ*stx*_*2dact*_), which is deficient in phage excision and replication (Table 3; [76]), produced levels of Stx_*2dact*_ indistinguishable from wild type (Fig 2). Apparently, enhanced expression of late genes *stx*_*2dact*_*A* and *stx*_*2dact*_*B* still occurs in the absence of integrase and is sufficient for wild type levels of Stx_*2dact*_ production. As expected, antiterminator protein Q, required for the transcription of late genes including *stx*, was essential for Stx_*2dact*_ production by *C*. *rodentium* (Φ*stx*_*2dact*_), consistent with previous findings for the Stx2 phage Φ361 [26]. Finally, the S endolysin of Stx phage H-19B was previously shown to promote the timely release of toxin after mitomycin C induction [27]; we found that deficiency of the RS endolysins encoded by Φ*stx*_*2dact*_ appeared to diminish the release of Stx_*2dact*_ into culture supernatants at 4 hours post-induction (Fig 2B). However, the decrease was not statistically significant, and RS-deficiency had no discernible effect on toxin release by 16 hours (Fig 2C). Dead and dying *E*. *coli* cells are known to release their contents into the surroundings at the end of stationary phase [84]; additionally, *E*. *coli* O157:H7 has been shown to release Shiga toxin via outer membrane vesicles [85].

Whereas previous work in streptomycin-treated or gnotobiotic murine models has demonstrated that induction of the lytic developmental program of Stx phage occurs during infection and is required for disease [40, 41, 69], we document here that prophage induction occurs during infection of mice with intact microbiota. *attP* sequences (indicative of excised, uningegrated phage genomes) were detected in the feces of infected mice, as revealed by deep sequencing (Table 3), or by qPCR (Fig 3).

Deep sequencing of phage genomes in the stool of mice revealed no evidence of Φ*stx*_*2dact*_ lysogeny of commensal bacteria during *C*. *rodentium*(Φ*stx*_*2dact*_) murine infection, suggesting that secondary infection of commensals by this phage is rare. Furthermore, *C*. *rodentium*(Φ*stx*_*2dact*_) mutants deficient in phage integrase or portal protein B, which retained the ability to produce Stx_*2dact*_, but were incapable of generating phage or infecting commensal bacteria, caused weight loss and lethality of mice with kinetics indistinguishable from wild type *C*. *rodentium*(Φ*stx*_*2dact*_) (Fig 4). Indeed, the only *C*. *rodentium*(Φ*stx*_*2dact*_) derivatives incapable causing disease in animals were those with a demonstrated defect in the production of Stx_*2dact*_ *in vitro* (Table 3 and Fig 4). For example, RecA, essential for the initiation of the SOS response that leads to prophage induction, was required for lethality after oral inoculation of *C*. *rodentium* (Φ*stx*_*2dact*_), consistent with the previous finding that RecA was required for lethality following intravenous EHEC infection of conventional mice [74]. We conclude that amplification of Stx_*2dact*_ production by successive rounds of lytic infection of commensal bacteria, as has been postulated [38, 44, 45, 47], is not required for toxin-mediated disease in this microbiota-replete model.

We detected more than 1 x 10^9^ phage/ml in uninduced mid-log cultures, suggesting that there is a high level of spontaneous induction under *in vitro* culture conditions. In contrast, despite severe Stx_*2dact*_-mediated disease manifestations during productive infection by *C*. *rodentium*(Φ*stx*_*2dact*_), the number of *attP* sequences detected in feces was extremely low, suggesting that the level of prophage induction during infection may also be low. On day 6 post-infection, only 0.44% of all phage genomes detected were excised, compared to 99.66% that were integrated, reflecting intact prophage (Table 3). Depending on the day post-infection, excised phage detected by qPCR numbered 20- to 1000-fold fewer than viable *C*. *rodentium*(Φ*stx*_*2dact*_) cells (Fig 3). Notably, previous work using a genetic reporter to indicate activation of lytic promoters of EHEC Stx phage 933W showed that the intestinal environment of a gnotobiotic mouse was strongly inducing [41]. While we cannot rule out the possibility that the low number of Φ*stx*_*2dact*_ *attP* sequences detected in feces reflects an instability of phage particles or some other factor in the intestinal milieu, our findings are consistent with the possibility that a low rate of Φ*stx*_*2dact*_ induction may be sufficient to promote disease in this model. Given that the methods to measure phage particles utilized in this study can be applied to patient samples, future studies will focus on the extent of lytic induction of Stx phage during human infection, and how it may correlate with disease outcome.

## Materials and methods

### Ethics statement

Mice were purchased from Jackson Laboratories and maintained in the Tufts University animal facility. All procedures were performed in compliance with Tufts University IACUC protocol B2014-87. If examination revealed signs of suffering, manifested by greatly diminished activity, poor grooming/appearance, biting, greatly increased respiratory rate or diminished appetite, or weight loss greater than 15% of body weight, then the animal was euthanized. Primary euthanasia method: CO2 asphyxiation or CO2 followed by cardiac stick. Secondary euthanasia method. Cervical dislocation, decapitation, thoracotomy or major organ removal is performed following the primary method."

### Bacterial strains and plasmids

Strains and plasmids used in this study are listed in Table 1.

### Phage Φ*stx*_*2dact*_ whole genome sequencing, assembly, and integration site determination

Genomic DNA was isolated from 5 ml of strain *C*. *rodentium*(Φ*stx*_*2dact*_::*kan*^*R*^) (Table 1) grown overnight at 37°C in LB broth containing chloramphenicol (12.5 μg/ml) and kanamycin (25 μg/ml). DNA was extracted using a DNeasy kit (Qiagen), according to the manufacturer’s protocol for Gram negative bacteria. A library of this DNA was then constructed for Illumina sequencing using Illumina TruSeq DNA Sample Preparation Kit per the manufacturer’s instructions. Following sequencing, the bacterial genome was assembled *de novo* into 1500 contigs using assemblers ABySS [86], and Edena [87]. The Bowtie2 program [88] was then used to map the *stx2* gene against this assembled genome and the contig containing this gene was identified. When aligned to the *C*. *rodentium* genome, a 69594-bp contig revealed a 47,343 bp prophage containing the *stx2* gene and other phage lambda-like gene sequences inserted into the host *dusA* gene. (Although the *C*. *rodentium dusA* gene is interrupted by the prophage genome, a potentially functional *dusA* gene is reconstituted at the *attL* bacterial/phage DNA junction by fusion with a prophage-derived open reading frame that we term “Φ*dusA’”* in Fig 1.) The prophage sequence was deposited in GenBank as Φ1720a-02, accession number KF030445.1.

Integration of the prophage in both *C*. *rodentium*(Φ*stx*_*2dact*_) and *C*. *rodentium* (ΦΔ*stx*_*2dact*_::*kan*^*R*^) into the host *dusA* gene was verified by PCR amplification of the *attL* and *attR* phage-host junctions using primers DusF/PhageR and DusR/PhageF, respectively (Table 4), then DNA sequencing of the amplified junctions. Subsequent whole genome sequencing of *C*. *rodentium*(Φ*stx*_*2dact*_) and *C*. *rodentium*(ΦΔ*stx*_*2dact*_::*kan*^*R*^) showed that, except for the Φ*stx*_*2dact*_ prophage sequences, they are identical to *C*. *rodentium* ICC 168, also known as strain DBS100 (GenBank accession number NC_013716.1), and to each other. The encoded Φ*stx*_*2dact*_ prophage sequences were identical except for the presence of the *kan*^R^ gene in *stxA* of strain *C*. *rodentium* (ΦΔ*stx*_*2dact*_::*kan*^*R*^) (S1 Fig) flanked by the sequence TCCCCGGGTCATTATTCCCT CCAGGTA upstream of the *kan*^R^ gene and the sequence CTTATTCCTCCTAGTTAGTCACCCGGGA downstream of the *kan*^R^ gene.

**Table 4:** Primers used in this study.

Primer	-------->
**Primers for Mutant Construction and Validation**	
*Cr* (Φ*Δ SR*) F	ATCGGTGTGTGCCGGTGGTCTTTATATTGTTGTGAGCTTCC GGATTGCGGGAGACGGGGTGGTCATGATCAGCACGTGTT GACAATTAATCATCGG
*Cr* (Φ*Δ SR*) R	CAGCCCATAACAGACAGACGATGATGCAGATAACCAGAG CGTAAATAATCGCGGTTACTCTTCTCAGTCCTGCTCCTCG GCCACGAAGTGCACGCAG
*Cr* (Φ*Δ SR*) validation F	CAACGAGAAAATCCCATGTCAGAAATTACATCCCTGGTC
*Cr* (Φ*Δ SR*) validation R	CTCATCAGCTTACTCTCCCCGCGCCGC
*Cr* (Φ*Δ int*) F	CGTTAGGTTCCCGCACAGGTTCCCACGTTTTATGGGAACC CGAAATAACGAGGTCGTGTAGGTCATGATCAGCACGTGTT GACAATTAATCATCGG
*Cr* (Φ*Δ int*) R	ATACTGTGTTTGTATACAGTATCATTTTTAACTGTATGGATA AACAGTGTCAGTCCTGCTCCTCGGCCACGAAGTGCACGC AG
*Cr* (Φ*Δ int*) validation F	GGGAACCCGAAATAACGAGGTCGTGTA
*Cr* (Φ*Δ int*) validation R	CATTTTTAACTGTATGGATAAACAGTG
*Cr* (Φ*Δ Q*) F	AGTAACCACTCTTAACATACTGACATACTTTTTGCGGACC GCGCTAATCATTTTGGTCATGATCAGCACGTGTTGACAATT AATCATCGG
*Cr* (Φ*Δ Q*) R	CGTTTTATCGATCGCGCGCTGGCGATTGGTGTGCTGTCCT GATTTTGTGGAGAAAGTTGTCAGTCCTGCTCCTCGGCCAC GAAGTGCACGCAG
*Cr* (Φ*Δ Q*)500bpextn F	ACCAGCCGCCCATTTACCAC
*Cr* (Φ*Δ Q*)500bpextn R	CCGGAAAGTGCAGCCCGTAAG
*Cr* (Φ*Δ Q*) validation F	TGCGGACCGCGCTAATCATTTT
*Cr* (Φ*Δ Q*) validation R	CCTGATTTTGTGGAGAAAGTTG
Q100 R	CGGATACCGTGGCATTTGA
*Cr* (Φ*Δ B*) F	GCCGCGATGGTGAGCCGCAGGCGGGGAAAACCGGGATT TAAACTGGCGAGGTTTTAGGTCATGATCAGCACGTGTTGA CAATTAATCATCGG
*Cr* (Φ*Δ B*) R	TCGTCATAAATATAAATATCCGCGTCACCCGGCCCCCCA GCCTGCATCCTGAACCAGGATTCAGTCCTGCTCCTCGGC CACGAAGTGCACGCAG
*Cr* (Φ*Δ B*) validation F	ACCGGGATTTAAACTGGCGAGGTTTTA
*Cr* (Φ*Δ B*) validation R	CCCCCCAGCCTGCATCCTGAACCAGGAT
*Cr* (Φ)*Δ recA* F	AATTGCTTCAACAGTACAGAATTCACTATCCGGATAAGCG CAAGCGGAACCCGGCATGACAGGAGTAGTTAGGTCATGA TCAGCACGTGTTGACAATTAATCATCGG
*Cr* (Φ)*Δ recA* R	ACCCTGAGTTGTAACTTACCTTCTTGCCGGACGGCAGCTT TGCGCCATCCGGCTTGCGGTTACCTGAAAATCAGTCCTG CTCCTCGGCCACGAAGTGCACGCAG
*Cr* (Φ)*Δ recA* validation F	ACTGTATGAGCATACAGTAT
*Cr* (Φ)*Δ recA* validation R	GCAAAAGGGCCGCATAAGCG
*Cr*(Φ)*Δ qseC* F	CTGGGCAGCGATTTTATTCGTACCGTTCACGGCATCGGCT ATACCCTTAGCGAGGCATAAAAGGTCATGATCAGCACGTG TTGACAATTAATCATCGG
*Cr*(Φ)*Δ qseC* validation F	ACGCCGTTGAGGTTCACGTCC
*Cr*(Φ)*Δ qseC* validation R	GCAAAATGCGTTTGAGGCT
*Δ* ROD24971 F	GTGTTCCTGTTTTAGTCGCGTAACCGGTTGCTAACCGTATC ATATCTTGCGGTATGTTGCGGAGGGTCATGATCAGCACGT GTTGACAATTAATCATCGG
*Δ* ROD24971 R	ACACGCCTGACGCGATACACGGTGATGACCACCCCGCCG CGCCGGTATCGCCTGACGAAGAGGTATCTCAGTCCTGCTC CTCGGCCACGAAGTGCACGCAG
*Δ* ROD24971 validation F	GGTTTAATAATCGCATCAATC
*Δ* ROD24971 validation R	CGTAAGCCAGGCGGGAGCTAC
*Cr* (Φ)*Δ rpoS* F	CGCAGCGATAAATCGACGGAGCAGGCTGACACGGGCTTG TTTTGTCAAGGGATCACGGGTAGGAGCCACCTTGGTCATG ATCAGCACGTGTTGACAATTAATCATCGG
*Cr* (Φ)*Δ rpoS* R	AGCGGGCAATAATGCAGCCAAAGAAAAAGACCAGCCTCAC AGAGACTGGTCTTTTCTGATGGAACGGTGCTCAGTCCTGC TCCTCGGCCACGAAGTGCACGCAG
*Cr* (Φ)*Δ rpoS* validation F	ATAGCGACTATGGGTAGCAC
*Cr* (Φ)*Δ rpoS* validation R	CCCGCCAGATCTGATAAGCG
**PCR and qPCR Primers**	
AttP F	CTTTGGATAGGTTCCCAATAGGC
AttP R	GGGTTCCCATAAAACGTGGG
RecA F	CGCTGACGTTACAGGTGATCGC
RecA R	CCATAGAGGATCTGGAACTCGG
Dus F	CCTTCGGGCTAAGCCCGG
Dus R	GCGCCGTCCACGCGAGG
Phage F	GTGACCAAGGCGTACCTGGC
Phage R	CCATCACTTTCTGTGTGCCCC
**Primers for Construction of Sequencing Library**	
PCR Primer 1	TTGCTTTCCCTGTAAGTGATAACACC
PCR Primer 2	GTGACTGGAGTTCAGACGTGTGCTCTTCCGATCTGGGGG GGGGGGGGGGG
PCR Primer 3	AATGATACGGCGACCACCGAGATCTACACTCTTTTTTACTG GAATTCTCGGTTTAGCATTGCTCCT
PCR Primer 4	CAAGCAGAAGACGGCATACGAGATTAAGGCGAGTGACTGG AGTTCAGACGTGTGCTCTTCCGATCT
Seq-P	ATCTACACTCTTTTTTACTGGAATTCTCGGTTTAGCATTGCT Cct

Cr = Citrobacter rodentium

### Phage Φ*stx*_*2dact*_ genome annotation

The Φ*stx*_*2dact*_ genome sequence was first annotated using the program RAST (http://rast.nmpdr.org/ [89]). The annotation was further refined by analyzing each open reading frame using the NCBI program MEGABLAST against the GenBank nucleotide database. Note that although the insertion of the marker into the *Rz*, gene affects lysis by phage λ lysogens in the presence of high magnesium, this gene has been altered in other studies of Stx phage [66] and in this study, lysis of *C*. *rodentium* (Φ*stx*_*2dact*_) occurred upon *in vitro* induction (S4 Fig).

### Characterization of phage and prophage sequences in murine stool by massively parallel sequencing and analysis

DNA was extracted from fecal samples of 5 infected sick mice at 6 days post-infection, according to the method of Yang et al. [90]. Twenty mg stool samples were suspended in 5 ml PBS, pH7.2, and centrifuged at 100 × g for 15 min at 4°C. The supernatant was centrifuged at 13,000 × g for 10 min at 4°C, and the resulting pellet was washed 3 times in 1.5 ml acetone, centrifuging at 13,000 × g for 10 min at 4°C after each wash step. Two hundred μl of 5% Chelex-100 (Bio-Rad) and 0.2 mg proteinase K were added to the pellet and the sample was incubated for 30 min at 56°C. After vortexing briefly, the sample was centrifuged at 10,000 × g for 5 min and the supernatant containing the DNA was harvested and stored.

To characterize bacteria that harbor the Φ*stx*_*2dact*_ prophage, we sequenced the bacterial bacterial-host *attL* prophage junction and adjacent bacterial DNA by following, with slight modifications, the methodology of Klein et al. [70] for constructing high-throughput sequencing libraries that contain a repetitive element (in this case, the phage *int* (integrase) gene). Briefly, genomic DNA was sheared by sonication to a size of 100–600 bp, followed by addition of ~20 deoxycytidine nucleotides to the 3’ ends of all molecules using Terminal deoxynucleotidyl Transferase. Two rounds of PCR using a poly-C-specific and phage *int* gene-specific primer pair (PCR primers 1 and 2, Table 4) were used to amplify *attL* and to add on sequences necessary for high-throughput sequencing (PCR primers 3 and 4, Table 4).

Amplicons were sequenced using the MiSeq desktop sequencer (Ilumina) and primer Seq-P (Table 4), providing reads of up to 300 bp. As amplicons spanned the region from the phage *int* gene, through *attL*, and into the adjacent host genome (see Fig 1), reads of this length were required. 17,868,095 sequences encompassing 5 Gb were downloaded to the Galaxy server (https://usegalaxy.org/) and analyzed (Table 3). We first excluded sequences that clearly reflected *attL* (i.e., contained the 184 bp of Φ*dusA’* followed by *C*. *rodentium dusA*), indicating the prophage inserted into the *C*. *rodentium* genome. Of the remaining 801,959 sequences, 75,962 (0.44% of the total) encoded the intact *attP* site, implying that they were circular. These latter sequences presumably reflected excised circular phage genomes, possibly undergoing early theta DNA replication, ultimately leading to phage production. The remaining 725,997 sequences encoded only strings of A’s and/or C’s, and were eliminated from consideration.

### Generation of *C*. *rodentium*(Φ*stx*_*2dact*_) deletion constructs

Deletion mutants of *C*. *rodentium*(Φ*stx*_*2dact*_) in the prophage or the host genome were generated using a modified version of a one-step PCR-based gene inactivation protocol [61, 82]. Briefly, a PCR product of the zeocin-resistance gene and its promoter region flanked by 70–500 bp homology of the region upstream and downstream of the targeted gene was generated using the primers listed in Table 4. The chromosomal DNA served as template when the flanking regions were 500 bp in length on either side of the zeocin cassette. The PCR product was electroporated into competent *C*. *rodentium*(Φ*stx*_*2dact*_) cells containing the lambda *red* plasmid pKD46 and recombinants were selected on plates containing chloramphenicol and zeocin (75 μg/ml). Replacement of the gene of interest with the zeocin resistance cassette was confirmed using specific primers (Table 4). At least two independent clones, validated using PCR, were obtained and subsequently analyzed.

To complement *C*. *rodentium*(Φ*stx*_*2dact*_*ΔQ*), the only phage mutant with a defect in Stx_*2dact*_ production, the region encoding the anti-terminator Q was amplified from WT genomic DNA using primers Cr (ΦΔQ) validation F and Q100 R (Table 4), cloned into the pCR4-TOPO vector and transformed into Top 10 cells using the TOPO TA cloning kit (Life Technologies). Kanamycin-resistant colonies were screened for the presence of vector carrying the *Q* gene (pTOPO-Q). pTOPO-Q was then transformed into electrocompetent wild type *C*. *rodentium*(Φ*stx*_*2dact*_) or *C*. *rodentium*(Φ*stx*_*2dact*_ *ΔQ*), using standard cloning techniques.

### Quantification of Stx_2dact_ produced *in vitro*

Overnight 37°C cultures of *C*. *rodentium*(Φ*stx*_*2dact*_) or deletion derivatives were diluted 1:25 into 10 ml of fresh medium with appropriate antibiotics. Two independently derived clones for each mutant were tested, with indistinguishable results. The cultures were grown at 37°C with aeration to an OD_600_ of 0.4, and one ml of each culture was set aside (Table 3, “t = 0h”). The remaining culture was split into 2 cultures. These cultures were grown for a further 4 hours (Table 3, “t = 4h”) in the absence or presence of 0.25 μg/ml mitomycin C. (We first measured phage and Stx2 production at various times post-induction and found the 4-hour time point to be optimal for obtaining maximal phage and Stx2 following mitomycin C induction). Supernatants depleted of intact bacteria were then harvested by centrifugation at 17,800 **×** g for 5 minutes at room temperature. For *C*. *rodentium*(Φ*stx*_*2dact*_) and *C*. *rodentium* (Φ*stx*_*2dact*_*ΔSR*), a portion of each culture was also collected after ~16 h of incubation (“t = 16h”). Supernatants and pellets were quantitated for Stx_*2dact*_ by ELISA, as described previously [52].

### Quantification of phage genomes by qPCR

Attempts to quantitate phage using plaque titers were unsuccessful. Attempts included the use of various host strains, including *C*. *rodentium* non-lysogens, *E*. *coli* K12 strains Epi300, LE392, or DH5α, *E*. *coli* OP50, and *Shigella*. Plate modifications included the addition of subinhibitory concentrations of antibiotics, addition of 10 mM CaCl_2_ or MgCl_2_ or both, addition of 5% glycerol to bottom agar, addition of tetrazolium to bottom agar, or Sybr staining and fluorescence microscopy of phage. Instead, excised phage genomes in cell supernatants were quantitated by qPCR. Protease digestion of capsids prior to qPCR quantitation was not required, as capsid undergoes melting during the high heating steps of the PCR procedure [75]. Supernatants were serially diluted 1:10, 1:100 and 1:1000 in distilled water. Separate reactions using two μl of the various dilutions as a template were carried out in duplicate. qPCR master-mix (Bio-Rad) was prepared according to the manufacturer’s instructions, using the *attP* primer set (Table 4) to detect copies of excised phage DNA. Results were compared to a standard curve, derived from a known concentration of a template fragments generated from amplifying *C*. *rodentium(*Φ*stx*_*2dact*_*)* DNA using *attP* primers. The template was serially diluted, in duplicate, to detect copy numbers ranging from 10^10^ to 10^2^. qPCR reactions were carried out as follows: 95°C for 3 min, followed by 35 cycles of 95°C for 1 min, 58°C for 30 sec, and 72°C for 1 min. Phage genomes in the supernatant of *C*. *rodentium* (Φ*stx*_*2dact*_*ΔB*), which lacks the portal protein required for genome packaging, was diminished 18-fold by DNAse treatment, supporting our method of qPCR quantitation of phage (see Table 3).

### Mouse infection studies

Mice were purchased from Jackson Laboratories and maintained in the Tufts University animal facility. Seven to eight-week-old female C57BL/6J mice were gavaged with PBS or ∼5×10^8^ CFU of overnight culture of *C*. *rodentium*(Φ*stx*_*2dact*_) or deletion derivatives in 100 μl PBS. Inoculum concentrations were confirmed by serial dilution plating. Fecal shedding was determined by plating dilutions of fecal slurry on either chloramphenicol, to detect wild type *C*. *rodentium*(Φ*stx*_*2dact*_), or chloramphenicol-zeocin plates, to detect deletion derivatives marked with a zeomycin resistance gene [52]. Body weights were monitored daily, and mice were euthanized upon losing >15% of their body weight.

DNA from infected mouse fecal pellets was isolated using the QIAGEN DNeasy Blood and Tissue kit with modifications. Fecal pellets were incubated with buffer ATL and proteinase K overnight at 55°C. Buffer AL was added, and after mixing, pellets were further incubated at 56°C for 1 h. Pellet mixtures were then centrifuged at 8000 rpm for 1 min and the pellets were discarded. Ethanol was added to the supernatants, which were processed according to the manufacturer’s protocol. DNA concentrations were determined using a NanoDrop spectrophotometer. qPCR was performed as described above.

### Statistical tests

Data were analyzed using GraphPad Prism software. Comparison of multiple groups were performed using the Kruskal-Wallis test with Dunn's multiple comparison post-test, or 2-way ANOVA with Bonferroni’s post-tests. In all tests, P values below 0.05 were considered statistically significant. Data represent the mean ± SEM in all graphs.

## Supporting information

S1 Fig *C*. *rodentium*(Φ*stx*_*2dact*_) prophage annotation. The 47,239 bp prophage DNA sequence (gray), flanked by *attL* and *attR* upon insertion into *C*. *rodentium dusA* sequence (blue, “*Cr dusA*”), was determined by whole genome shotgun sequencing of *C*. *rodentium* (Φ*stx*_*2dact*_::*kan*^*R*^) and annotated, as described in Materials and Methods. Names of encoded proteins are shown. Unannotated ORFs indicate hypothetical proteins. At the far left end is a phage sequence that encodes the N-terminal 112 amino acids of an open reading frame (“Φ*dusA’*”) in the same reading frame as the 3’ end of the *C*. *rodentium dusA* gene. Strain *C*. *rodentium*(Φ*stx*_*2dact*_) encodes a chloramphenicol acetyl transferase protein (“*cat*”) inserted into the prophage *Rz* gene. The sequence of *C*. *rodentium*(Φ*stx*_*2dact*_::*kan*^*R*^) is identical to *C*. *rodentium* (Φ*stx*_*2dact*_) except that the gene encoding the A subunit of Stx_*2dact*_ (“Stx2A”) contains an 894 bp insertion encoding kanamycin resistance (“*kan*”), plus an additional 27 bp upstream and 28 bp downstream. Prophage genes studied in this work are shown in bold. *Cr*: *C*. *rodentium*.
(TIFF)

S2 Fig Comparison of λ, 933W, Sp5 and 1720a-02 prophage maps. The location of the host integration site and genome size for each prophage is indicated in parentheses after the phage name. Open reading frames of prophages λ, 933W, and Sp5 [28, 91, 92] are shown in comparison to those of prophage 1720a-02, and are depicted as arrows pointing in the direction of transcription. The site of insertion of the chloramphenicol cassette (*cat*) in phage 1720a-02 is indicated with an open triangle. The dotted blue line indicates the presence of additional prophage genes. Genes and genomes are not drawn to scale.
(TIF)

S3 Fig Regions of homology between phage Φ(*stx*_*2dact*_) and the *C*. *rodentium* wild type genome. The sequence of phage Φ*stx*_*2dact*_ was used as a query to interrogate the *C*. *rodentium* DBS100 genome (i.e., the parent of the Φ*stx*_*2dact*_ lysogen) for regions of homology, using the program Megablast (NCBI). Two regions of homology, each to a different endogenous *C*. *rodentium* prophage, were identified. **A.** Region of homology between Φ*stx*_*2dact*_ and a hypothetical protein. **B**. Region of homology encompassing a gene encoding a hypothetical protein (upstream of *cro*), the *cro* gene (demarcated by red arrows), and a large portion of the *cI* gene, (demarcated by a blue arrow). Black arrows indicate the direction of transcription.
(TIFF)

S4 Fig *C*. *rodentium*(Φ*stx*_*2dact*_) lyses following induction with mitomycin C. Cultures were grown in LB medium to OD_600_ = 0.4 (T = 0), then each was divided into two cultures. One culture was induced with mitomycin C (0.25 μg/ml) and the other was left uninduced. OD_600_ culture readings were followed for 4 hours. Two independent isolates of strain *C*. *rodentium* (Φstx_*2dact*_*ΔQ*) (“*ΔQ*”), both unable to produce large bursts of phage on induction, were used as the control. Black arrow indicates time at which mitomycin C was added.
(TIFF)

S5 Fig Φ(*stx2*_*dact*_) infects and lysogenizes *E*. *coli* K12 strain DH5α. Lysogens of strain DH5α were obtained by infecting a log phase culture with Φ*stx*_*2dact*_ at high multiplicity of infection, according to the method of Ray and Sakalka [93], and lysogens were isolated by selecting for kanamycin-resistant survivors. A. Agarose gel of PCR analysis showing that a putative DH5α(Φ*stx*_*2dact*_) lysogen and *C*. *rodentium* control lysogens encode Φ*stx*_*2dact*_ genes *SR*, whereas a DH5α non-lysogen did not. B. Strains DH5α containing the *recA*-bearing plasmid pER271, and the same strain harboring the Φ*stx*_*2dact*_ prophage, were streaked on LB plates. One half of the plate was shielded with aluminum foil (-UV), while the unprotected half (+UV) was illuminated for 15 seconds using a UVP model UVGL-25 Mineralight UV lamp at 254 nm wavelength from a distance of 8 inches, then incubated overnight at 37°C in the dark.
(TIF)

S6 Fig *C*. *rodentium*(Φ*stx*_*2dact*_) mutants display no growth defects in LB or DMEM medium. The indicated wild type or mutant *C*. *rodentium* (Φ*stx*_*2dact*_) strains were grown in LB broth or DMEM (Gibco, GlutaMAX) without antibiotics. Growth was measured over time by optical density (OD_600_), and growth curves are the average of duplicate samples. Doubling times were calculated based on the exponential growth regions of each curve. Representative results from one of two experiments are shown.
(TIF)

S7 Fig Complementation of the ΔQ mutation partially restores Stx_*2dact*_ levels *in vitro*. Q-deficient *C*. *rodentium*(Φ*stx*_*2dact*_Δ*Q*) (“Δ*Q*”), *C*. *rodentium*(Φ*stx*_*2dact*_Δ*Q*)/pTOPO-Q (“Δ*Q+*pQ”), wild type *C*. *rodentium*(Φ*stx*_*2dact*_)/pTOPO-Q (“*“*WT+pQ”) and wild type *C*. *rodentium*(Φ*stx*_*2dact*_) (“WT") were grown to mid-log phase and cultured for four more hours either in the absence (“-“) or presence (“+”) of 0.25 μg/ml mitomycin C. Pellets (filled bars) or supernatants (open bars) were subjected to capture ELISA to determine the level of Stx_*2dact*_ production. Quantities are expressed relative to the specific OD_600_ at t = 0h. Results are averages ± SEM of triplicate samples, and are a representative of one of two experiments. nd, not detected. Asterisks indicate Stx levels significantly (p<0.05) different from *C*. *rodentium*(Φ*stx*_*2dact*_Δ*Q*) calculated using Kruskal–Wallis one-way analysis of variance followed by Dunn's nonparametric comparison.
(TIFF)

S8 Fig QseC, QseF, and RpoS are not required for wild type basal and induced levels of Stx_*2dact*_ production *in vitro*. The indicated lysogens were grown to mid-log phase (designated as t = 0h) and cultured for four more hours (t = 4h) either in the absence (“-“) or presence (“+”) of 0.25 μg/ml mitomycin C. Pellets (filled bars) or supernatants (open bars) were subjected to capture ELISA to determine the level of Stx_*2dact*_ production. Quantities are expressed relative to the specific OD_600_ at t = 0h. Results are averages ± SEM of triplicate samples, and are a representative of at least two experiments. Stx levels of the *C*. *rodentium qseC*, *qseF*, or *rpoS* mutant strains were not significantly different from wild type *C*. *rodentium*(Φ*stx*_*2dact*_), calculated using Kruskal–Wallis one-way analysis of variance followed by Dunn's multiple comparisons test.
(TIF)

S9 Fig *C*. *rodentium*(Φ*stx*_*2dact*_) mutants do not display colonization defects. Eight-week old female C57BL/6 mice were infected by oral gavage with the indicated lysogens. Fecal shedding of the lysogens was determined by plating for viable counts (see Materials and Methods). No significant differences were observed, as determined by 2-way ANOVA. **A.** Colonization of mice by wild type or *recA*- or prophage mutant lysogens. **B.** Colonization of mice by wild type or quorum-sensing mutant lysogens.
(TIF)

S10 Fig QseC and RpoS are not required for disease by *C*. *rodentium*(Φ*stx*_*2dact*_). Eight-week old female C57BL/6 mice were infected by oral gavage with the indicated lysogens. **A.** Percentage weight change was determined at indicated post-infection time. Data shown are averages ± SEM of 10 mice per group. No significant differences were observed, as determined by 2-way ANOVA. **B.** Percent survival at the indicated post-infection time was monitored in 10 mice per group. Data represent cumulative results of 3 separate experiments.
(TIF)
