# Supplementary figures and images for: Prophage induction, but not production of phage particles, is required for lethal disease in a microbiome-replete murine model of enterohemorrhagic E. coli infection
Source: PLoS Pathog. 2019 Jan 10;15(1):e1007494. doi: 10.1371/journal.ppat.1007494 (PMC6328086; doi:10.1371/journal.ppat.1007494)

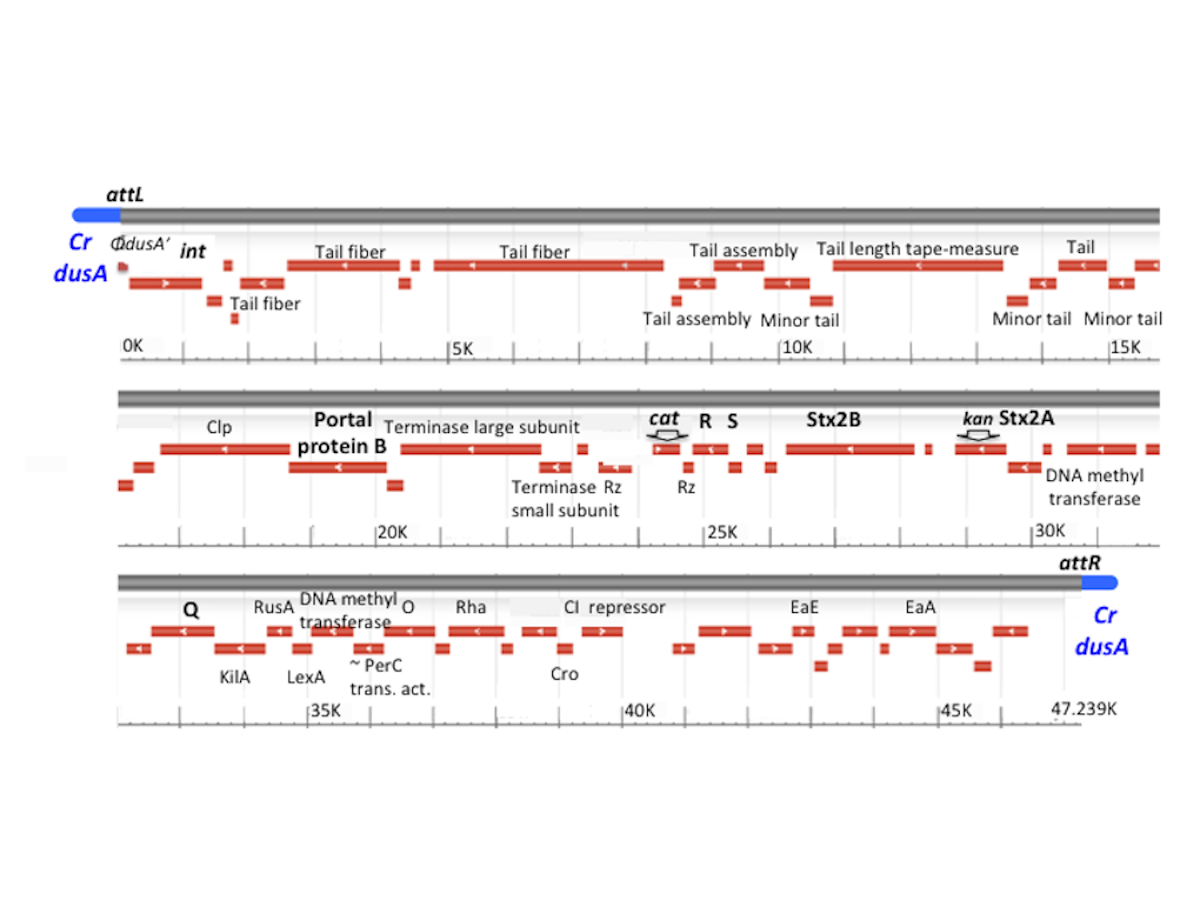

Supplement: S1 Fig — The 47,239 bp prophage DNA sequence (gray), flanked by attL and attR upon insertion into C. rodentium dusA sequence (blue, “Cr dusA”), was determined by whole genome shotgun sequencing of C. rodentium (Φstx2dact::kanR) and annotated, as described in Materials and Methods. Names of encoded proteins are shown. Unannotated ORFs indicate hypothetical proteins. At the far left end is a phage sequence that encodes the N-terminal 112 amino acids of an open reading frame (“ΦdusA’”) in the same reading frame as the 3’ end of the C. rodentium dusA gene. Strain C. rodentium(Φstx2dact) encodes a chloramphenicol acetyl transferase protein (“cat”) inserted into the prophage Rz gene. The sequence of C. rodentium(Φstx2dact::kanR) is identical to C. rodentium (Φstx2dact) except that the gene encoding the A subunit of Stx2dact (“Stx2A”) contains an 894 bp insertion encoding kanamycin resistance (“kan”), plus an additional 27 bp upstream and 28 bp downstream. Prophage genes studied in this work are shown in bold. Cr: C. rodentium. (TIFF) [file ppat.1007494.s001.tiff]

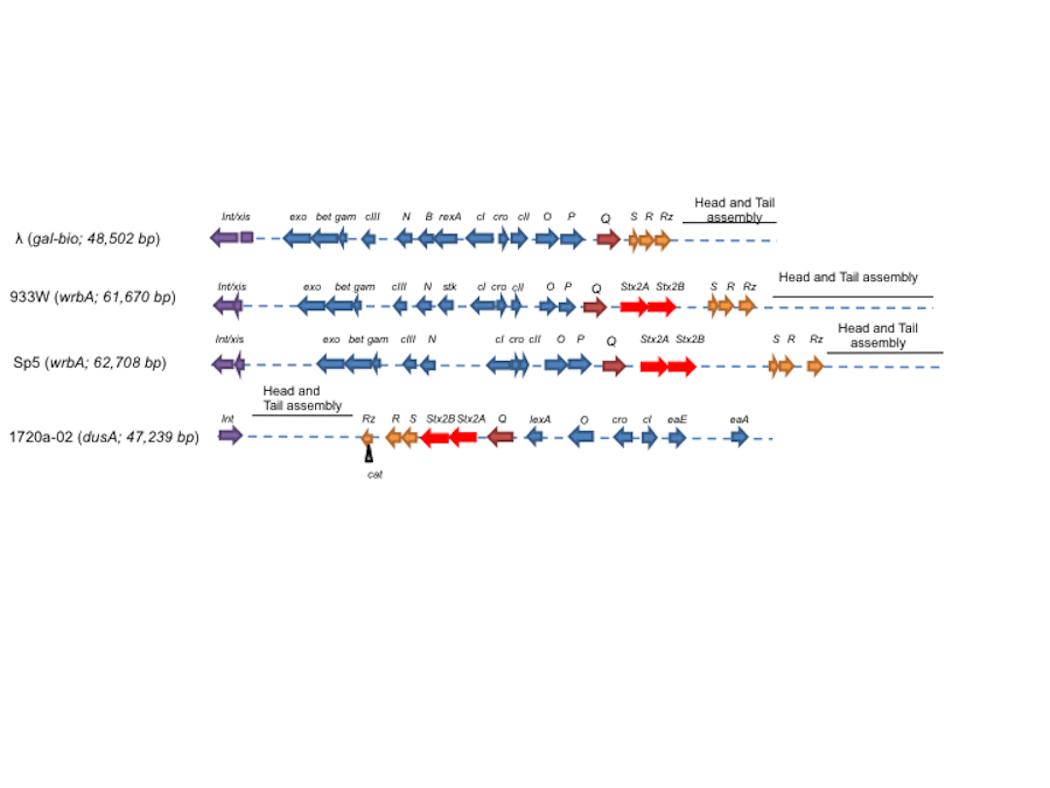

Supplement: S2 Fig — The location of the host integration site and genome size for each prophage is indicated in parentheses after the phage name. Open reading frames of prophages λ, 933W, and Sp5 [28, 91, 92] are shown in comparison to those of prophage 1720a-02, and are depicted as arrows pointing in the direction of transcription. The site of insertion of the chloramphenicol cassette (cat) in phage 1720a-02 is indicated with an open triangle. The dotted blue line indicates the presence of additional prophage genes. Genes and genomes are not drawn to scale. (TIF) [file ppat.1007494.s002.tif]

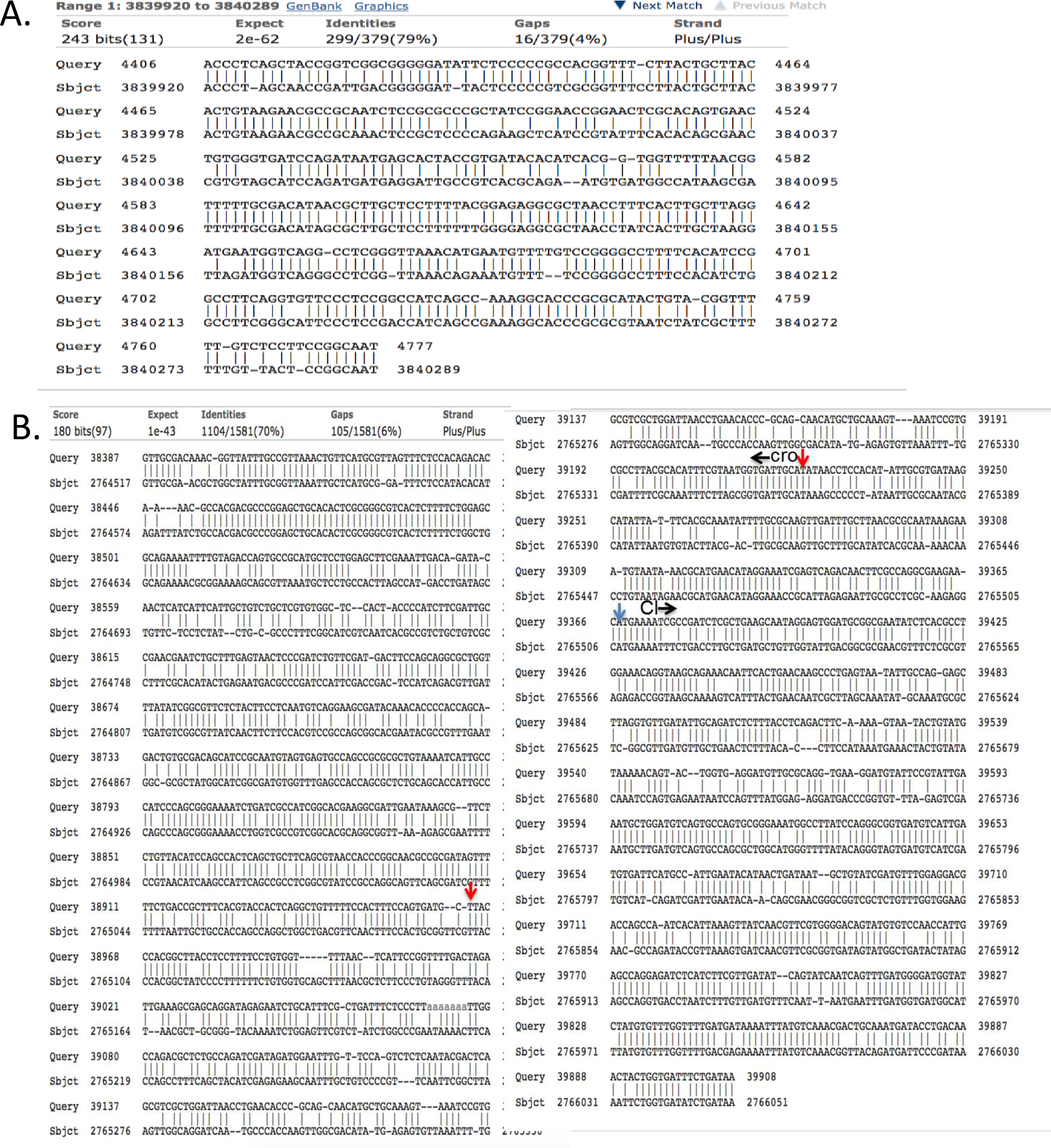

Supplement: S3 Fig — The sequence of phage Φstx2dact was used as a query to interrogate the C. rodentium DBS100 genome (i.e., the parent of the Φstx2dact lysogen) for regions of homology, using the program Megablast (NCBI). Two regions of homology, each to a different endogenous C. rodentium prophage, were identified. A. Region of homology between Φstx2dact and a hypothetical protein. B. Region of homology encompassing a gene encoding a hypothetical protein (upstream of cro), the cro gene (demarcated by red arrows), and a large portion of the cI gene, (demarcated by a blue arrow). Black arrows indicate the direction of transcription. (TIFF) [file ppat.1007494.s003.tiff]

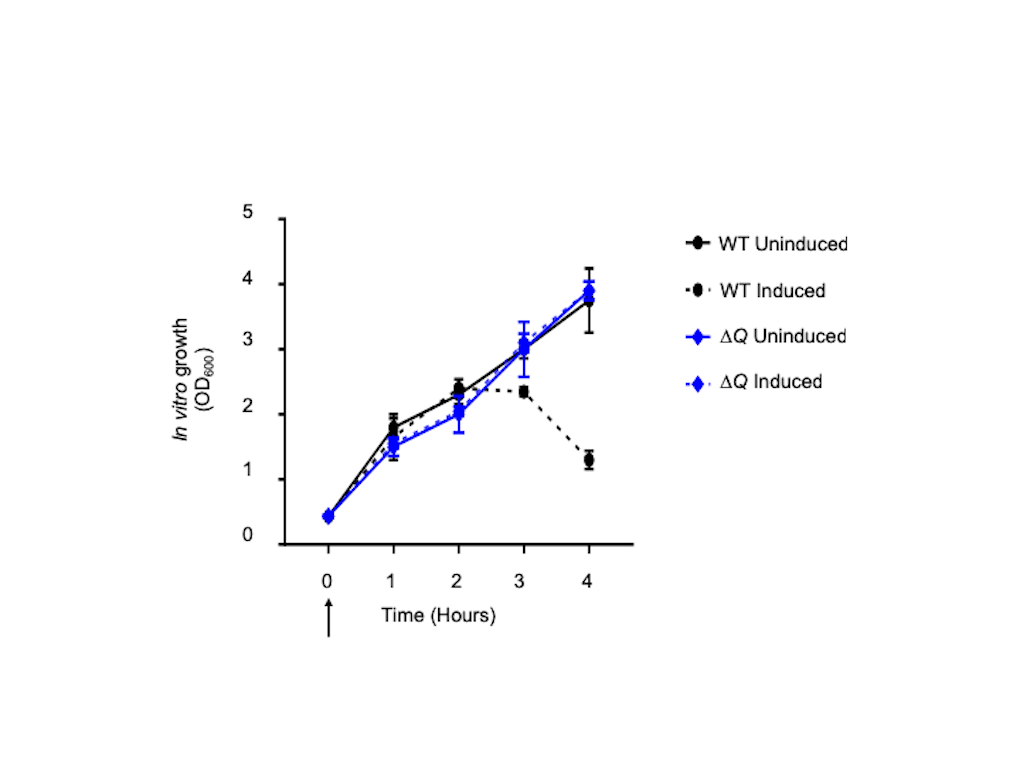

Supplement: S4 Fig — Cultures were grown in LB medium to OD600 = 0.4 (T = 0), then each was divided into two cultures. One culture was induced with mitomycin C (0.25 μg/ml) and the other was left uninduced. OD600 culture readings were followed for 4 hours. Two independent isolates of strain C. rodentium (Φstx2dactΔQ) (“ΔQ”), both unable to produce large bursts of phage on induction, were used as the control. Black arrow indicates time at which mitomycin C was added. (TIFF) [file ppat.1007494.s004.tiff]

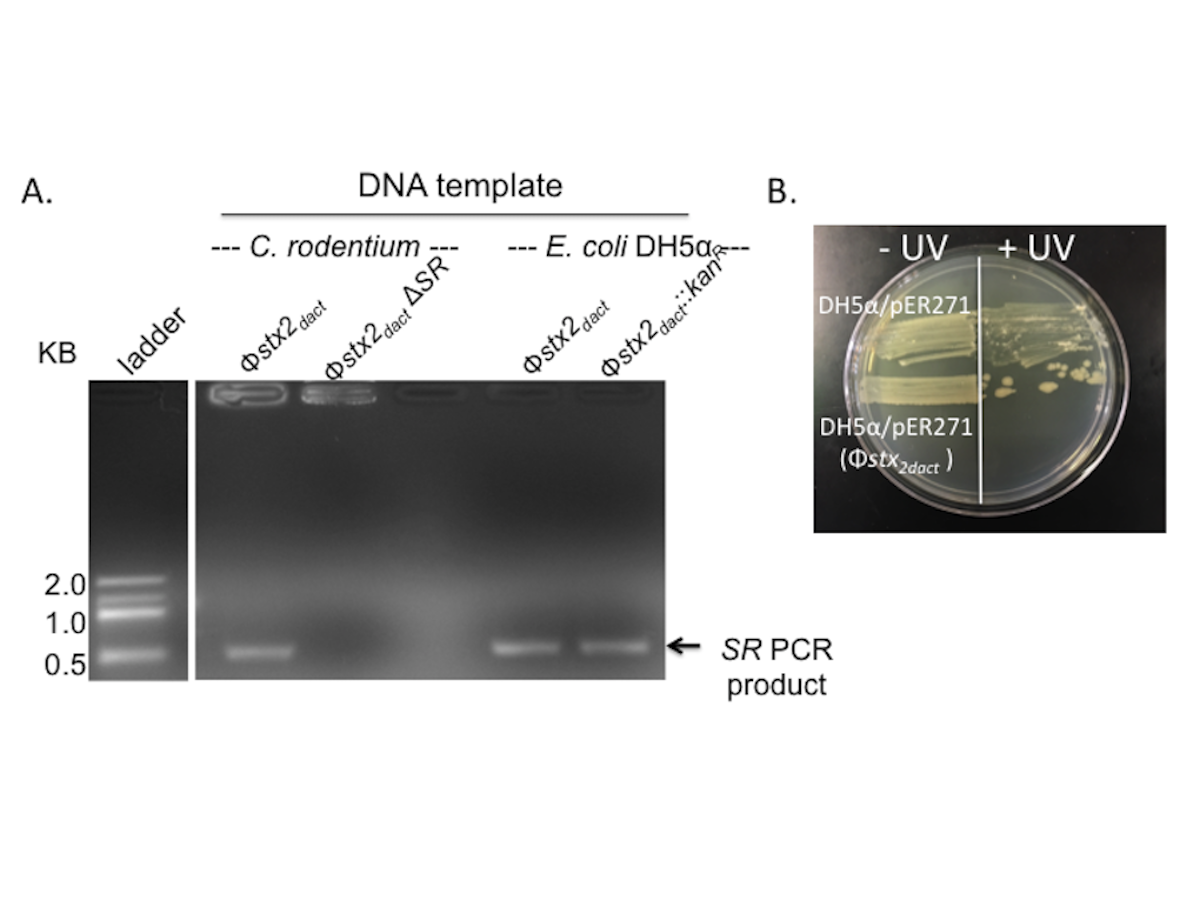

Supplement: S5 Fig — Lysogens of strain DH5α were obtained by infecting a log phase culture with Φstx2dact at high multiplicity of infection, according to the method of Ray and Sakalka [93], and lysogens were isolated by selecting for kanamycin-resistant survivors. A. Agarose gel of PCR analysis showing that a putative DH5α(Φstx2dact) lysogen and C. rodentium control lysogens encode Φstx2dact genes SR, whereas a DH5α non-lysogen did not. B. Strains DH5α containing the recA-bearing plasmid pER271, and the same strain harboring the Φstx2dact prophage, were streaked on LB plates. One half of the plate was shielded with aluminum foil (-UV), while the unprotected half (+UV) was illuminated for 15 seconds using a UVP model UVGL-25 Mineralight UV lamp at 254 nm wavelength from a distance of 8 inches, then incubated overnight at 37°C in the dark. (TIF) [file ppat.1007494.s005.tif]

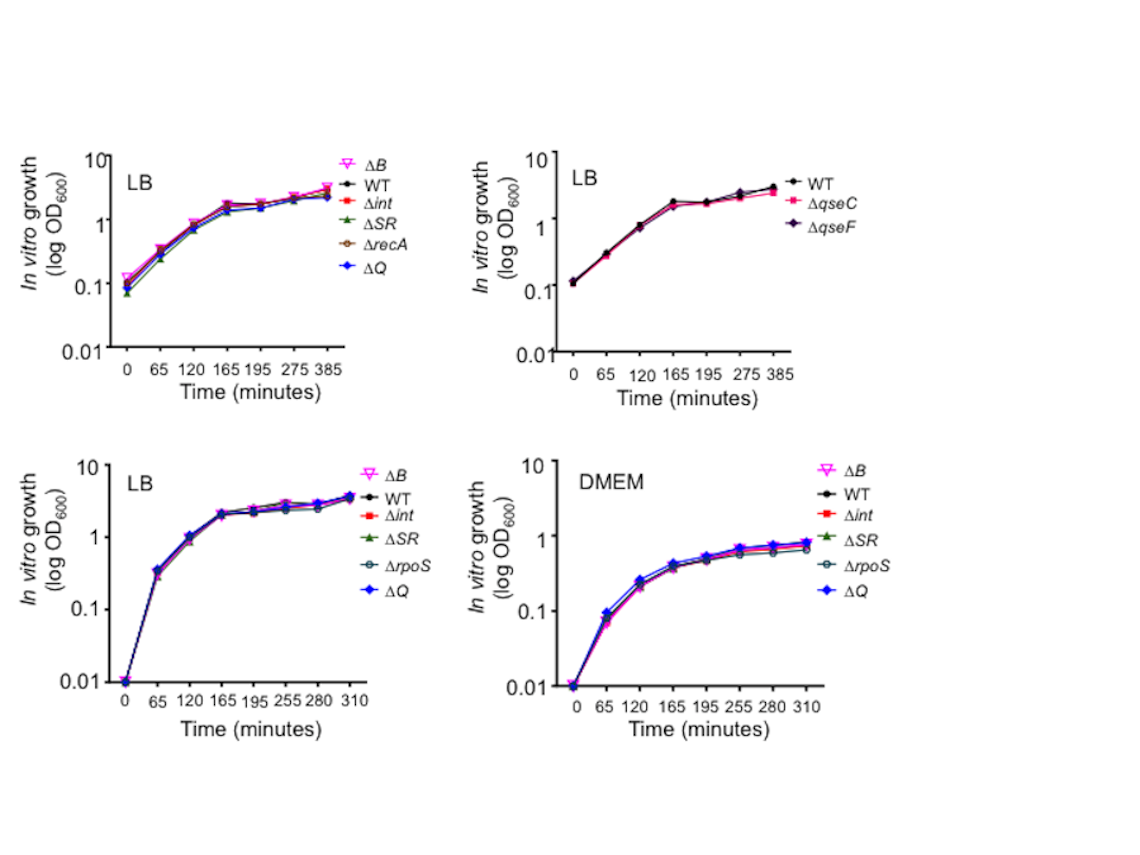

Supplement: S6 Fig — The indicated wild type or mutant C. rodentium (Φstx2dact) strains were grown in LB broth or DMEM (Gibco, GlutaMAX) without antibiotics. Growth was measured over time by optical density (OD600), and growth curves are the average of duplicate samples. Doubling times were calculated based on the exponential growth regions of each curve. Representative results from one of two experiments are shown. (TIF) [file ppat.1007494.s006.tif]

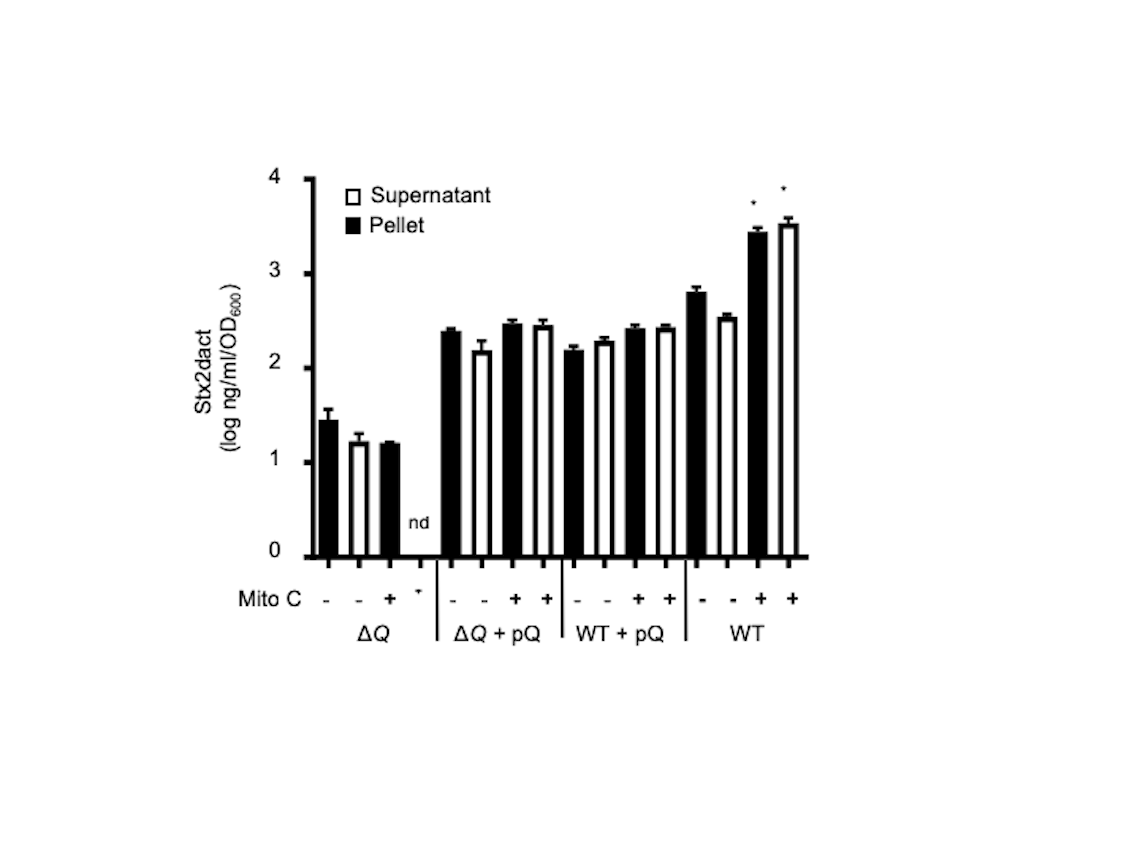

Supplement: S7 Fig — Q-deficient C. rodentium(Φstx2dactΔQ) (“ΔQ”), C. rodentium(Φstx2dactΔQ)/pTOPO-Q (“ΔQ+pQ”), wild type C. rodentium(Φstx2dact)/pTOPO-Q (““WT+pQ”) and wild type C. rodentium(Φstx2dact) (“WT") were grown to mid-log phase and cultured for four more hours either in the absence (“-“) or presence (“+”) of 0.25 μg/ml mitomycin C. Pellets (filled bars) or supernatants (open bars) were subjected to capture ELISA to determine the level of Stx2dact production. Quantities are expressed relative to the specific OD600 at t = 0h. Results are averages ± SEM of triplicate samples, and are a representative of one of two experiments. nd, not detected. Asterisks indicate Stx levels significantly (p<0.05) different from C. rodentium(Φstx2dactΔQ) calculated using Kruskal–Wallis one-way analysis of variance followed by Dunn's nonparametric comparison. (TIFF) [file ppat.1007494.s007.tiff]

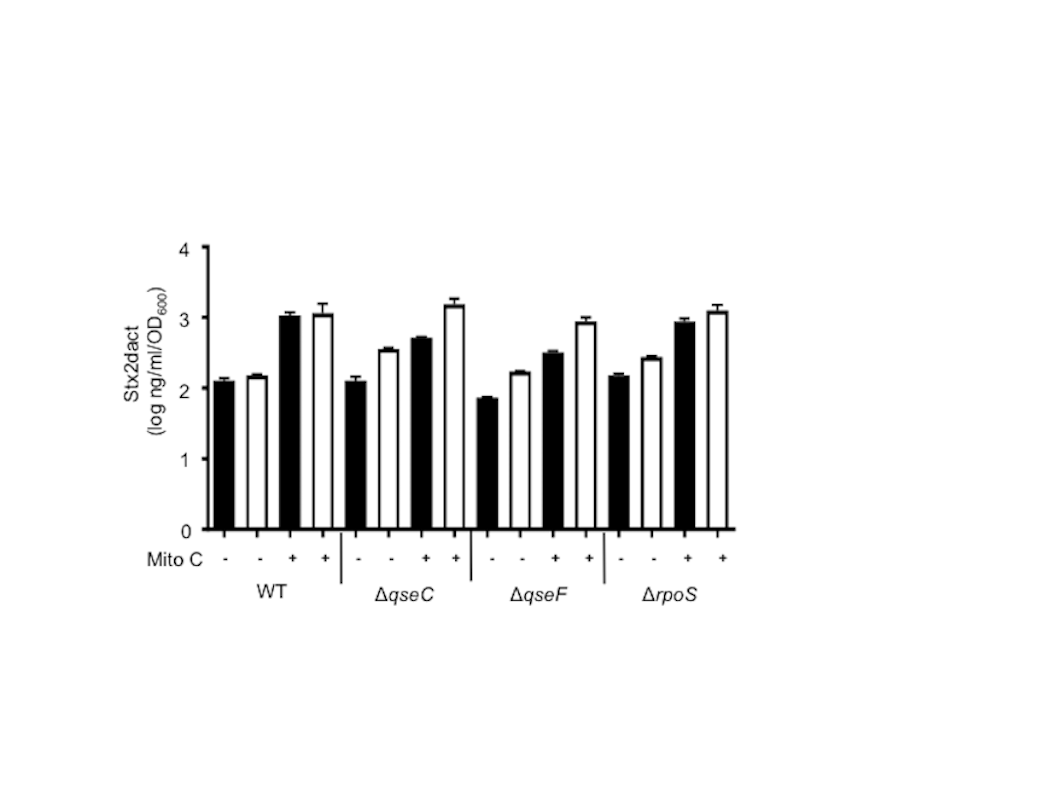

Supplement: S8 Fig — The indicated lysogens were grown to mid-log phase (designated as t = 0h) and cultured for four more hours (t = 4h) either in the absence (“-“) or presence (“+”) of 0.25 μg/ml mitomycin C. Pellets (filled bars) or supernatants (open bars) were subjected to capture ELISA to determine the level of Stx2dact production. Quantities are expressed relative to the specific OD600 at t = 0h. Results are averages ± SEM of triplicate samples, and are a representative of at least two experiments. Stx levels of the C. rodentium qseC, qseF, or rpoS mutant strains were not significantly different from wild type C. rodentium(Φstx2dact), calculated using Kruskal–Wallis one-way analysis of variance followed by Dunn's multiple comparisons test. (TIF) [file ppat.1007494.s008.tif]

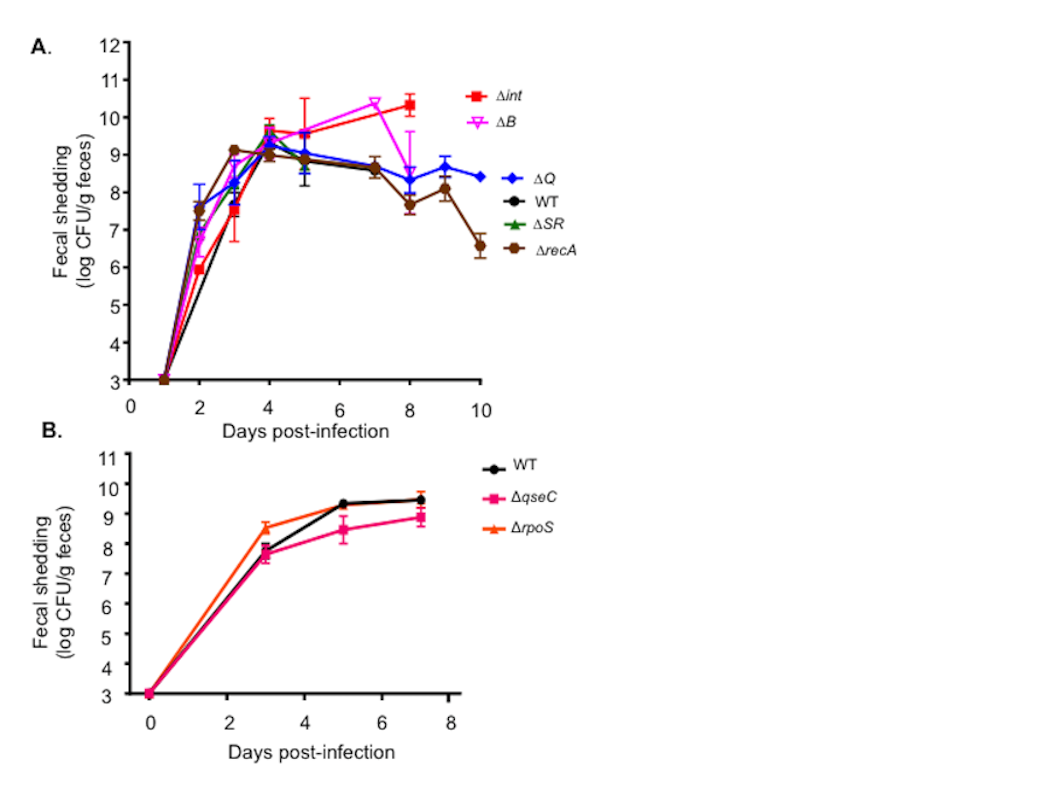

Supplement: S9 Fig — Eight-week old female C57BL/6 mice were infected by oral gavage with the indicated lysogens. Fecal shedding of the lysogens was determined by plating for viable counts (see Materials and Methods). No significant differences were observed, as determined by 2-way ANOVA. A. Colonization of mice by wild type or recA- or prophage mutant lysogens. B. Colonization of mice by wild type or quorum-sensing mutant lysogens. (TIF) [file ppat.1007494.s009.tif]

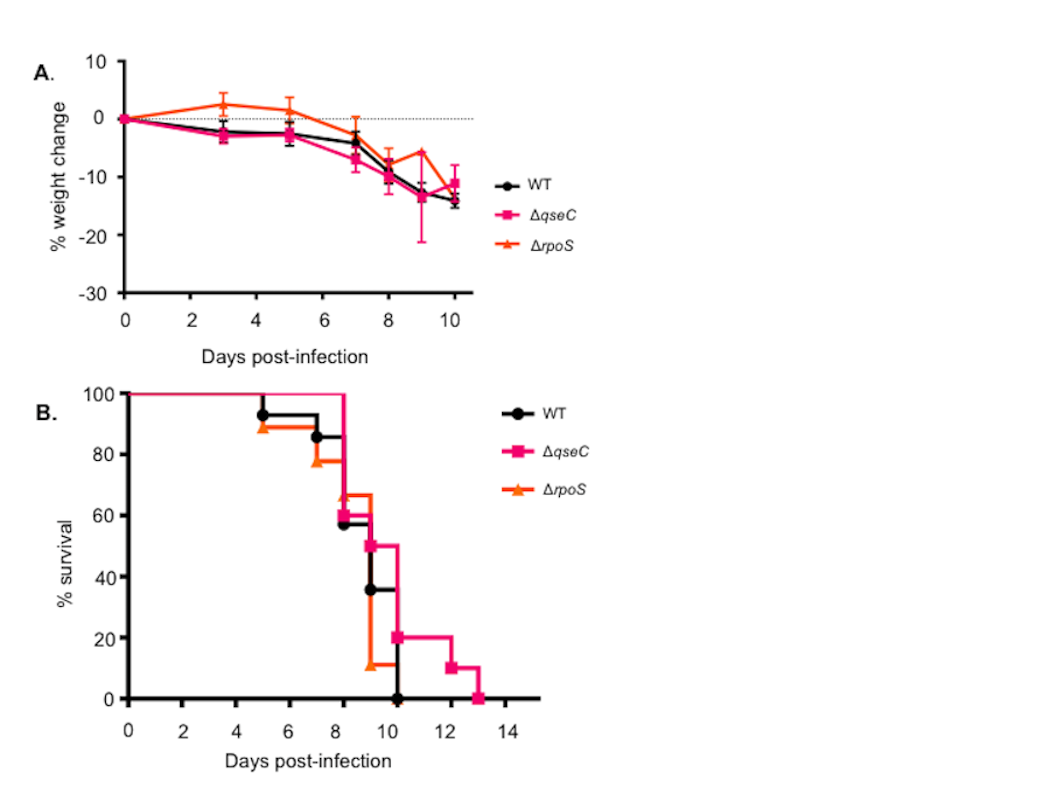

Supplement: S10 Fig — Eight-week old female C57BL/6 mice were infected by oral gavage with the indicated lysogens. A. Percentage weight change was determined at indicated post-infection time. Data shown are averages ± SEM of 10 mice per group. No significant differences were observed, as determined by 2-way ANOVA. B. Percent survival at the indicated post-infection time was monitored in 10 mice per group. Data represent cumulative results of 3 separate experiments. (TIF) [file ppat.1007494.s010.tif]
